# Epigenetic hereditary transcription profiles II, aging revisited

**DOI:** 10.1186/1745-6150-2-39

**Published:** 2007-12-28

**Authors:** Johannes WIM Simons

**Affiliations:** 1 Department of Toxicogenetics, MGC, Leiden University Medical Center, PO Box 9600, 2300, RC Leiden, The Netherlands

## Abstract

**Background:**

Previously, we have shown that deviations from the average transcription profile of a group of functionally related genes can be epigenetically transmitted to daughter cells, thereby implicating nuclear programming as the cause. As a first step in further characterizing this phenomenon it was necessary to determine to what extent such deviations occur in non-tumorigenic tissues derived from normal individuals. To this end, a microarray database derived from 90 human donors aged between 22 to 87 years was used to study deviations from the average transcription profile of the proteasome genes.

**Results:**

Increase in donor age was found to correlate with a decrease in deviations from the general transcription profile with this decline being gender-specific. The age-related index declined at a faster rate for males although it started from a higher level. Additionally, transcription profiles from similar tissues were more alike than those from different tissues, indicating that deviations arise during differentiation.

**Conclusion:**

These findings suggest that aging and differentiation are related to epigenetic changes that alter the transcription profile of proteasomal genes. Since alterations in the structure and function of the proteasome are unlikely, such changes appear to occur without concomitant change in gene function.

These findings, if confirmed, may have a significant impact on our understanding of the aging process.

**Open peer review:**

This article was reviewed by Nathan Bowen (nominated by I. King Jordan), Timothy E. Reddy (nominated by Charles DeLisi) and by Martijn Huynen. For the full reviews, please go to the Reviewers'comments section.

## Background

The 'creation' of Dolly, the cloned sheep [1] has proved that it is possible to reprogram the nucleus of a somatic cell and has suggested that there might be an epigenetic basis for differentiation and aging. Elucidating the underlying epigenetic structure is an unabated challenge.

In a recent study, deviation from the from the mean transcription profile of the 14 genes of a cellular organelle (the 20S proteasome) was arbitrarily chosen as a model system for assessing the degree of variation in transcription of a group of functionally related genes [2].

This analysis indicated that deviations from the mean transcription profile can be epigenetically transmitted to daughter cells [2]. When the 'mean transcription profile' was assigned as the norm, extensive deviations from this profile were found in many libraries. Not surprisingly, libraries from cancer tissues, as a group, showed more variation than the libraries from normal tissues. However, the unexpected finding that a deviating profile could be inherited, (in that it could be transmitted to a clonal derivative, for example, from a normal tissue to the tumor derived from this tissue) suggested the possible existence of epigenetic cellular structures that program the transcription of genes. "Epigenetic Hereditary Transcription Profiles" might therefore be a manifestation of the nuclear programming that is known to occur in cells and might be a tool in the study of nuclear programming.

Nuclear programming has become an important issue in animal cloning. The ability to clone organisms by transferring a nucleus from a somatic cell into an enucleated egg cell, has led to the notion that heritable epigenetic processes, that are in principle modifiable, control gene expression in differentiation and aging.

Although the nature of this non-DNA-based inheritance remains poorly understood it has been hypothesized that DNA methylation [3,4], chromatin organization by histone and non-histone architectural proteins [5,6], subnuclear organelles [7] and noncoding RNAs [8] are involved. Apparently the interplay of all these factors could result in unique assemblages that determine the accessibility of the underlying DNA. The relationship between these factors and carcinogenesis also underscores the importance of nuclear programming [9].

Apart from its significance in studies of animal cloning and carcinogenesis, nuclear programming could also be involved in exotic phenomena such as the trans-generational effects of parental irradiation [10], or of parental famine [11], reprogramming of tumor cells into normal cells [12,13] and might also support the evidence for epigenetic disruption as the first step in carcinogenesis [14,15].

It would also appear that elucidation of the processes underlying nuclear programming is likely to prove a crucial factor in improving our understanding of differentiation and cellular aging. Consequently it is conceivable that studying "Epigenetic Hereditary Transcription Profiles" will lead to further insights about the phenomenon of nuclear programming.

As a first step, therefore, in helping to elucidate the nature of these hereditary profiles, the current investigation was aimed at determining which factors modulate deviations from the mean transcription profile of the proteasomal genes in normal tissues.

It was observed that aging alters deviations from the mean proteasome transcription profile, with the data indicating a gender-specific difference. There also appeared to be some tissue specificity in deviations from the mean proteasome transcription profile, suggesting that the profile can be altered during differentiation. Additionally, further evidence for the occurrence of disturbed transcription profiles was obtained.

## Results and Discussion

### Proteasome expression level and age contribute to variation in transcription profile

The characteristics of the 90 libraries used for the analysis are shown in Table 1. The logarithms of the ratios of observed over expected transcript abundances (log obs/exp) are given in Additional file 1. These "log obs/exp" data were subjected to a Principal Component Analysis (PCA) to determine whether there were any major factors (in particular the two quantitative variables of the database, donor age and proteasome expression level) related to variability. Since the three most important factors accounted for only 31,2%, 18,2% and 15,7% of the variability observed, one cannot say that there is one major principal component involved.

**Table 1:** Characteristics of the 90 libraries

TISSUE	ARRAY NUMBER	SEX	AGE	SOURCE	HOURS	EXPRESSION LEVEL
					post mortem	
adrenal 1	shba125	Male	68	Autopsy	6,5	174,3
adrenal 2	shba136	Female		Surgical		75,6
adrenal 3	SHBW166	Male	62	Surgical		124,7
adrenal 4	shbw179	Female	87	Surgical		127,1
bladder 1	shbw160	Male	60	Surgical		78,0
bladder 2	shbw182	Male	83	Surgical		91,7
bowel 1	shbw184	Male	61	Surgical		94,4
bowel 2	shcn014	Male	60	Surgical		62,5
bowel 3	shcn056	Female		Surgical		88,0
brain 1	SHBW163	Male	60	Autopsy	71	90,9
brain 2	shbw196	Female	87	Autopsy	10	52,7
brain 3	shcn018	Female	54	Autopsy	24	94,7
brain 4	shbw195	Female	87	Autopsy	10	76,3
brain 5	shcn019	Female	54	Autopsy	24	91,4
brain 6	SHBW164	Male	60	Autopsy	71	73,8
brain 7	shbw197	Female	87	Autopsy	10	57,1
brain 8	shcn015	Female	54	Autopsy	24	55,5
breast	shba154	Female	36	Surgical		103,5
cervix 1	shbw158	Female	36	Surgical		78,8
cervix 2	shcn065	Female	72	Surgical		52,9
cervix 3	shcn074	Female	66	Surgical		67,4
colon 1	shbw155	Male	48	Surgical		67,2
colon 2	shcn069	Male	57	Surgical		63,1
colon 3	shcn070	Female		Surgical		60,8
diaphr	shbw150	Female		Surgical		203,6
epidyd	shcn076	Male	27	Surgical		75,9
esoph 1	shba117	Female		Surgical		121,6
esoph 2	shba151	Male	41	Autopsy	9,5	84,5
esoph 3	shba138	Female		Surgical		98,5
fallop t 1	shba118	Female		Unknown		83,0
fallop t 2	shba115	Female		Unknown		84,6
fallop t 3	shdp085	Female	72	Surgical		86,3
fallop t 4	shdp093	Female	42	Surgical		75,2
gallbl	shdp086	Male	63	Surgical		136,1
heart 1	shba147	Male	41	Autopsy	9,5	86,5
heart 2	shba150	Male	68	Autopsy	6,5	118,7
heart 3	shdp084	Female	46	Surgical		112,3
heart 4	shdp091	Female	23	Surgical		109,4
heart 5	shdp094	Male	54	Surgical		118,2
heart 6	shdp095	Male	47	Autopsy	32	82,9
kicney 3	shcn053	Male	50	Surgical		53,7
kidney 1	shbw194	Male	74	Surgical		49,0
kidney 2	shba122	Unknown		Surgical		90,3
kidney 4	shcn058	Female	65	Surgical		66,0
kidney 5	shcn059	Female	55	Surgical		65,6
liver 1	shba123	Male	68	Autopsy	6,5	132,8
liver 2	shba130	Male	32	Surgical		105,8
liver 3	shba132	Male	41	Autopsy	9,5	87,2
liver 4	shcn064	Male	73	Autopsy	10	115,0
liver 5	shcn066	Female	74	Surgical		120,4
lung 1	shbw168	Male	69	Surgical		86,5
lung 2	shbw175	Female	61	Surgical		58,1
lung 3	shbw183	Female	57	Surgical		58,3
lung 4	shba116	Female		Surgical		152,2
muscle 1	shba121	Male	41	Autopsy	9,5	197,3
muscle 2	shba155	Male	36	Surgical		149,0
ovary 1	shba137	Female		Surgical		70,8
ovary 2	shba139	Female		Surgical		75,4
ovary 3	shbw170	Female	63	Surgical		92,6
ovary 4	shbw187	Female	51	Surgical		74,7
ovary 5	shbw191	Female	56	Surgical		85,7
pancre 1	shba135	Female		Surgical		86,5
pancre 2	shcn051	Unknown		Surgical		77,9
pericard	shba141	Female		Surgical		79,3
placenta	shcn052	Female		Surgical		81,8
prostate 1	shbw152	Male	53	Surgical		107,4
prostate 2	shbw153	Male	59	Surgical		106,6
prostate 3	shbw154	Male	55	Surgical		75,8
prostate 4	shbw180	Male	82	Surgical		88,7
prostate 5	shba157	Male	76	Surgical		75,5
saliv gl 1	shbw151	Male	40	Surgical		74,2
saliv gl 2	SHBW165	Female	44	Surgical		77,6
saliv gl 3	shbw189	Male	60	Surgical		63,0
saliv gl 4	shbw149	Female	43	Surgical		124,9
sem ves 1	shcn084	Male		Surgical		92,8
sem ves 2	shcn081	Male		Surgical		92,6
sem ves 3	shcn077	Male		Surgical		121,0
stomach 1	shba120	Female		Surgical		87,0
stomach 2	shba134	Female		Surgical		61,4
stomach 3	shbw188	Female	62	Surgical		53,3
stomach 4	shcn057	Male	60	Surgical		79,3
testis 1	shba113	Male	68	Autopsy	6,5	123,1
testis 2	shbw181	Male	22	Surgical		119,9
testis 3	shcn055	Male	87	Surgical		96,3
uterus 1	shba114	Female		Surgical		106,8
uterus 2	shbw173	Female	53	Surgical		76,0
uterus 3	shbw178	Female	53	Surgical		59,9
uterus 4	shcn022	Female	62	Surgical		62,4
uterus 5	shbw159	Female	51	Surgical		90,9
vagina	shbw186	Female	46	Autopsy	23	49,7

However, a significant correlation between proteasome expression level (i.e. the total of transcript abundances found for all the proteasome genes in a given library) and a few of the factor loadings of the PCA: F2 (P = 2,65 × 10^-5^), F3 (P = 0,0016), F4 (P = 3,94 × 10^-5^) and F9 (P = 0,0039) was observed and together these factors are responsible for 46% of the variability.

Age also correlates with the loadings of some of the remaining factors: F1 (P = 0,016), F5 (P = 0,044) and F13 (P = 0,012), which cumulatively account for 40% of the variability. The higher P-values found for age could be due to the lower number of libraries, since age was known for only 68 of the 90 libraries.

Therefore both age and proteasome expression levels affect the proteasome transcriptional profile. The lack of correlation between proteasome expression level and age (P = 0,288) suggests that these factors exert independent effects within the database on the expression profile. The remainder of the analysis aims to visualize these effects.

### Proteasome expression level

As can be inferred from Table 1, there was an approximately 4-fold variation (49–203) in proteasome expression level. Since considerable variation will be due to regulation by transcription factors, variability in log obs/exp is only in part caused by epigenetic structures. Possibly the variation seen in proteasome expression level, seen in individual tissues, e.g. adrenals (75–174) and salivary glands (63–124), reflects largely variation due to gene regulation but the present data are not informative at this particular point.

To establish whether the variation in proteasome expression level affects the transcriptional profile of the proteasome, regression lines between the proteasome expression level and the "log obs/exp"of each individual gene-probe were calculated (Table 2).

**Table 2:** Regression lines between proteasome expression level (x) and "log obs/exp" (y) for all 90 libraries and after omission of 8 outlying libraries. Significant P-values are given in bold characters.

gene	90 libraries	probability	82 libraries	probability
A1	y = -0,0015x + 0,1476	0,00000	y = -0,0015x + 0,1509	0,00000
A2a	y = 0,0008x - 0,1225	0,27726	y = 0,0014x - 0,1648	0,03250
A2b	y = -0,0005x + 0,037	0,12799	y = -0,0004x + 0,0326	0,28234
A3a	y = -0,004x + 0,3336	0,00000	y = -0,0041x + 0,3225	0,00000
A3b	y = 0,0004x - 0,0481	0,68967	y = 0,0002x - 0,0179	0,60152
A4	y = -0,0015x + 0,0946	0,26356	y = -0,0014x + 0,145	0,00027
A5	y = 0,0014x - 0,1564	0,01839	y = 0,0012x - 0,1275	0,00084
A6	y = 0,001x - 0,1266	0,18951	y = 0,001x - 0,0985	0,02555
A7a	y = 0,0035x - 0,4248	0,00055	y = 0,0032x - 0,3946	0,00073
A7b	y = 0,0056x - 0,8918	0,05043	y = 0,0054x - 0,9019	0,04468
B2	y = 0,0012x - 0,1905	0,88566	y = 0,0004x - 0,0776	0,55212
B3	y = 0,0084x - 1,2972	0,00593	y = 0,0078x - 1,2842	0,01413
B5	y = 0,0096x - 1,1494	0,00005	y = 0,0066x - 0,8109	0,00003
B6	y = 0,0013x - 0,1999	0,34936	y = 0,001x - 0,1708	0,22870
B7a	y = 0,0045x - 1,1016	0,18447	y = 0,0059x - 1,2644	0,14417
B7b	y = 0,0045x - 1,1892	0,17417	y = 0,0049x - 1,2995	0,18850
B7c	y = -0,0086x + 0,2093	0,01378	y = -0,0089x + 0,2336	0,00587
B7d	y = 0,0048x - 0,5784	0,00034	y = 0,0045x - 0,5371	0,00021

The slopes of the regression lines differ widely. Three probes even have negative slopes, which means that tissues with a high proteasome expression level have less transcripts for these probes than tissues with a lower proteasome expression level, in other words: the transcriptional profile is different for tissues with different proteasome expression level*s*.

**Figure 1:**
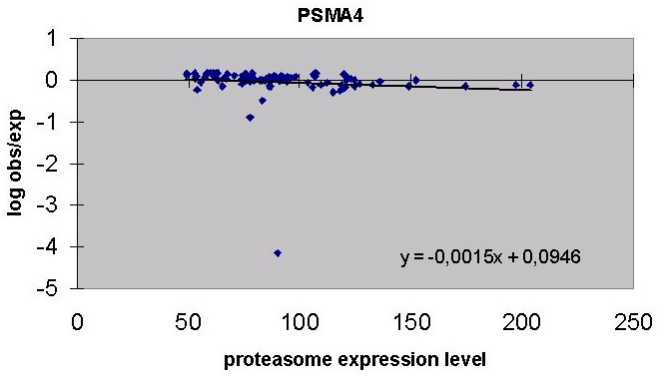
Regression of proteasome expression level with "log obs/exp"of PSMA4 (P = 0,263). All 90 libraries have been used. One library clearly falls outside the general range.

An example of a regression is shown for PSMA4 (Figure 1) and it can be seen that one library (kidney 2) falls well outside the general range and should therefore be considered an outlier. Some other gene probes have similar outliers; all representing strong under-expressions. In total, 8 libraries that deviated from the overall expression pattern were identified in this way. Seven of these deviated for 1 gene-probe with no preferential gene-probe being involved (liver 4, kidney 2, liver 2, heart 5, kidney 1, uterus 4 and ovary 4) and one library deviated for 5 gene-probes simultaneously (pancreas 2). These 8 outlying libraries were omitted from the subsequent analysis. Recalculation of the regression lines then revealed 4 probes with a significant negative slope (Table 2).

To determine the differences between genes in terms of absolute abundance of transcripts, regression lines were also calculated for proteasome expression level and absolute abundances (data not shown). These regression lines have been used to demonstrate the effect of proteasome expression level on the transcriptional profile of the proteasomal genes. Since Table 1 shows that the proteasome expression level lies within the range 49 – 203, the absolute abundances of transcripts at specific proteasome expression levels of 50 and 200 were calculated for each probe using its regression line (Table 3). This allows comparing the degree of transcription at the highest induction level (200) with the transcription at the lowest level (50). The figures differ for each probe but the response to high induction can be arbitrarily classified into 4 classes: some probes (B7c and A3a) are not induced and thus behave as constitutive genes (class A). Two probes (A4 and A1), already highly abundant at level 50, are enhanced about 2-fold at level 200. Other probes (A2a, A2b, A3b, A5, A6, B2, B6, B7a), which are moderately abundant at level 50 are induced to the expected 4-fold level (Class C), whilst others (A7a, A7b, B3, B5, B7b, B7d) which have extremely low levels of transcript abundance at level 50 are induced much more than 4-fold at level 200 (Class D).

**Table 3:** Types of upgrading of absolute expression when proteasome expression level increases from 50 to 200. Induction = (expr 200 - expr 50)/expr 200 ; the induction factor results from dividing this value by 0.75 (= expected value).

probe	transcript abundance		induction factor	induction type	induction class
	at expr 50	at expr 200			

B7c	1,16	0,29	0,00	no	A
A3a	2,76	2,50	0,00	no	A
A4	10,64	25,01	0,77	under	B
A1	11,89	28,30	0,77	under	B
A2b	4,84	16,62	0,94	expected	C
A3b	4,93	19,59	1,00	expected	C
B2	1,88	8,84	1,05	expected	C
A6	4,32	21,89	1,07	expected	C
A5	3,15	17,21	1,09	expected	C
A2a	2,37	13,27	1,10	expected	C
B7a	0,40	2,62	1,13	expected	C
B6	1,40	9,24	1,13	expected	C
B7b	0,18	1,71	1,19	over	D
B7d	0,47	7,76	1,25	over	D
A7a	0,29	11,43	1,30	over	D
B3	0,00	2,70	1,33	over	D
A7b	0,00	3,49	1,33	over	D
B5	0,00	7,55	1,33	over	D

One could therefore speculate that class D expression acts as a rate-limiting step in upgrading the number of proteasomes in the cell.

### Removal of the influence of proteasome expression level

In order to determine the contribution of variation in proteasome expression level to the variability in "log obs/exp", the regression lines from the 82 libraries (Table 2) were used to calculate expected values for "log obs/exp" which were then subtracted from the observed ones. The resulting values (provided in Additional file 2 and designated "2nd-log obs/exp") are free from the effect of proteasome expression level. Since the variance of "log obs/exp" is 0,3558 and that of "2nd-log obs/exp is 0,2629, it is apparent that about 26,1% (1-(0,2629/0,3558) = 0,261) of the total variability in "log obs/exp" is due to the level of expression of the proteasomal genes.

**Figure 2:**
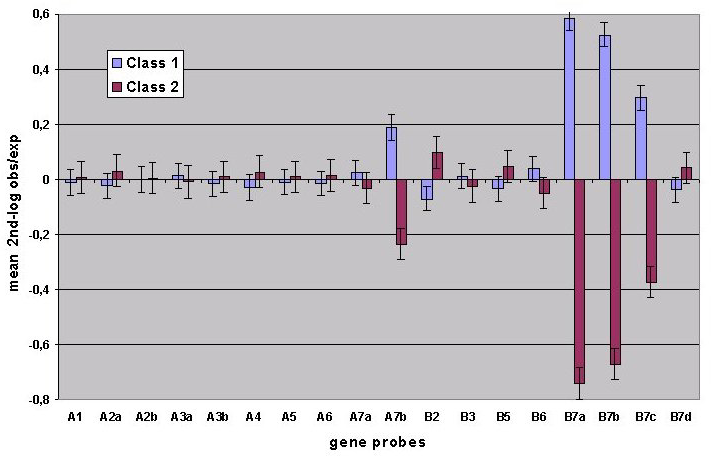
Mean profiles of 2nd-log obs/exp for the two classes obtained with k-means clustering.

**Figure 3:**
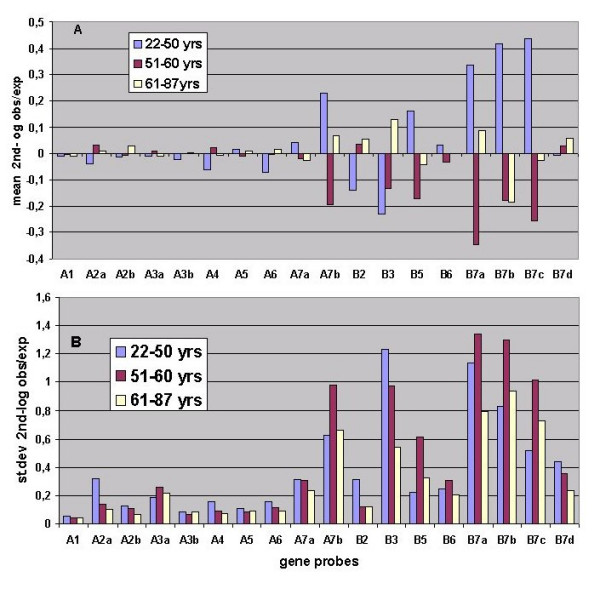
Mean (figure 3A) and standard deviation (figure 3B) of 2nd log obs/exp for three age groups.

The remaining variation in "2nd-log obs/exp" could be due either to factors associated with nuclear programming, such as gender, age and tissue specificity or to other factors such as post-mortem changes (if the biopsy was obtained by autopsy), 'noise' in transcription or experimental error. Also individual specificity in the transcription profile is, in theory, possible and belongs with the first factor type. To determine the possible influence of such factors, the libraries were divided into 2 classes by k-means clustering. The mean "2^nd ^log obs/exp", that indicate the deviations from expected transcription profiles, are depicted in Figure 2, with class 1 and class 2 showing major over-expressions or under-expressions respectively for 4 of the probes. These 4 probes belong to 2 genes and also belong to the group of 5 probes that were the most variable in "2nd-log obs/exp" (Figure 3B). The factors mentioned above were assessed for an eventual contribution to the variation in "2nd-log obs/exp" by comparing the numbers of factor variants in both classes and determining their probability by either a chi-square or Wilcoxon test. Since a comparison of the many variants in tissue specificity is not possible using this method, the mesodermic or endodermic origin of the tissues was used instead. The data in Table 4 demonstrate that with the exception of age (P = 3,64 × 10^-6^) none of these factors was significant, thereby suggesting that there is an increase in under-expression with age. The mean ages in classes 1 and 2 were 53,5 and 61,9 years respectively.

**Table 4:** Distribution of the factor variants over the two classes obtained with k-means clustering of 2^nd^-log obs/exp. Sex, biopsy and tissue were compared with chi-square, age and proteasome expression level with Wilcoxon.

Factor	variants	class 1	class 2	P-value
sex	male	18	18	0,432
	female	27	19	
biopsy	surgery	36	26	0,544
	autopsy	9	9	
tissue	endodermic	14	20	0.157
	mesodermic	19	21	
age	in years	53,5	61,9	3,64E-06
expression level		91,9	90,0	0,592

### Age-factor 1 (age related variation in mean transcript abundances)

**Figure 4:**
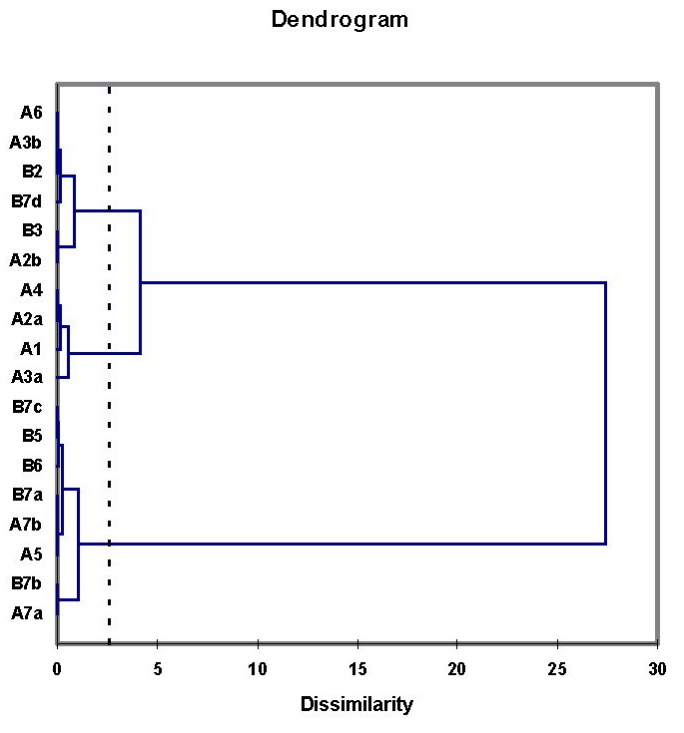
AHC of the mean 2nd-log obs/exp's from the three age groups identified three types of probes.

**Figure 5:**
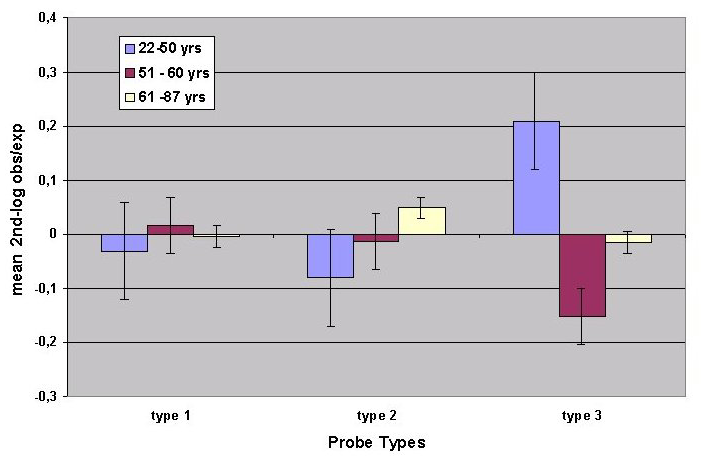
Mean 2nd-log obs/exp in the three age groups after pooling the data for the three types of probes.

**Figure 6:**
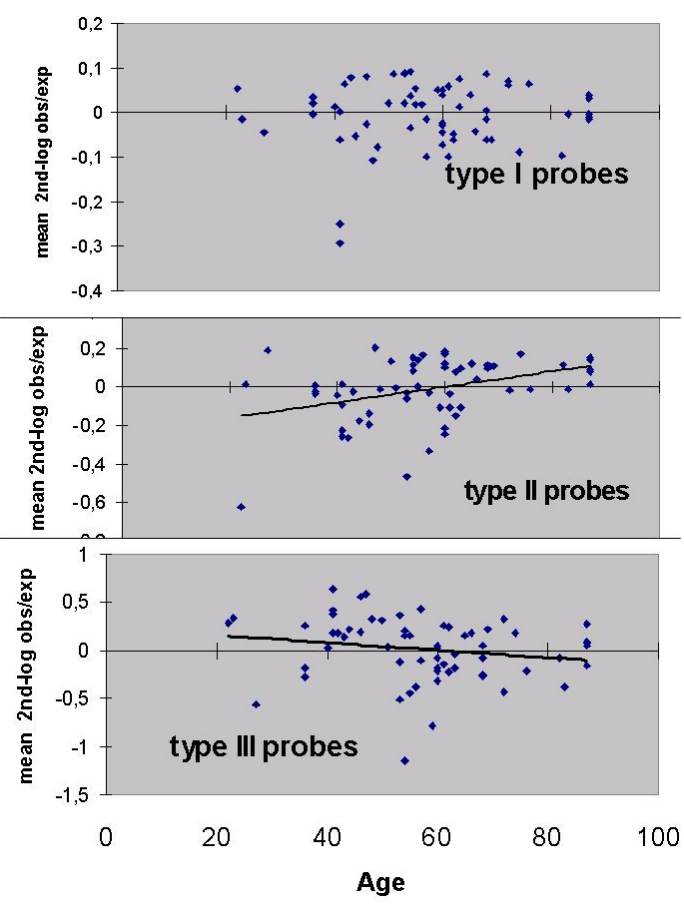
Relations with age of the 3 types of probes. Correlation coefficients are 0,001 0,43 and 0,27 respectively.

To further analyze the influence of aging, the libraries were assigned to 3 separate groups as follows: **A **(22 – 50 years, 19 libraries), **B **(51 – 60 years, 19 libraries) and **C **(61 – 87 years, 24 libraries). The mean and standard deviation of the "2nd-log obs/exp" for each age group are shown in Figures 3A and 3B respectively. A number of observations can be made from the data presented:

1) it appears that the standard deviations (Fig. 3B) may decrease with age, since when subjected to the Wilcoxon test a significant decrease was observed for group B versus C (P = 0,001) whereas the P-value of the decrease for A versus C was 0,094; 2) the standard deviations are much larger for some of the probes (A7b, B3, B5, B7a, B7b, B7c); 3) the same probes seem to show differences between the age groups with respect to mean "2^nd^-log obs/exp" and 4) the patterns of the mean "2^nd^-log obs/exp" over the three age groups appear different for different probes. This latter observation was investigated further by subjecting the age-group data from Figure 3A to agglomerative hierarchical clustering (AHC). This analysis (Figure 4) identified three probe-types, i.e. type 1: A1, A2a, A3a, A4; type II: A2b, A3b, A6, B2, B3, B7d, and type III: A5, A7a, A7b, B5, B6, B7a, B7b, B7c. Consequently the data for mean "2^nd^-log obs/exp" was pooled for each probe-type. The results, for the different age groups, shown in Figure 5 reveal that the three probe-types differ in relation to age. Therefore, for each probe-type, regressions were determined between age and the mean of the "2^nd^-log obs/exp" for the contributing probes (Figure 6). Although the regressions for type 1 probes for mean "2^nd^-log obs/exp" and mean abundance respectively, are not significant (P = 0,528 and P = 0,995), they are highly significant for type II probes (P = 0,0020 and P = 0,0006) and moderately significant for type III probes (P = 0,249 and P = 0,031.

It therefore appears that the type II and type III probes differ in their relation to age with type II showing an increase and type III a decrease in transcript abundance with aging. Two of the probes stand out in terms of a positive and negative correlation between age and transcript abundance, i.e. type II probe B2 (P = 0,020) and type III probe B7b (P = 0,009) respectively.

### Age-factor 2 (age related variation in residual values)

**Figure 7:**
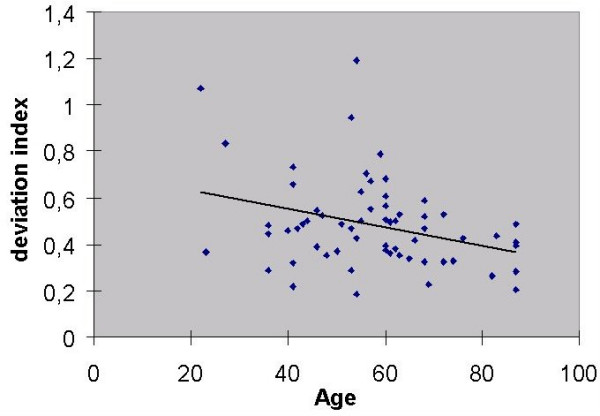
Deviation index (= standard deviation of all "2nd-log obs/exp's" in a library) decreases with age (P = 0,012). Correlation coefficient is 0,32.

To analyse the observed decrease in standard deviation (Fig. 3B) the relationship between age and the 'deviation index' was assessed. The 'deviation index' represents the standard deviation of all "2^nd^-log obs/exp" in a given library and indicates the extent to which the transcription profile of that library deviates from the mean standard transcription profile. It has previously been shown that this index is significantly enhanced in cancer tissues [2]. If the aging process results from the increasing accumulation of mutational damage one would expect to observe a parallel increase in the deviation index during aging. The opposite, however, appears to be the case, since the regression of age with the deviation index of "2^nd^-log obs/exp" (Figure. 7) shows that the deviation index decreases significantly with increasing age (P = 0,0117). This decrease might suggest that during aging, transcription becomes more accurate and/or less variable.

In order to determine whether all probes are similarly involved in this aging process, a new deviation index was first calculated in which the data from one probe was omitted and then the regression with age was recalculated. The rationale for this was that if a probe contributes to the aging effect, the P-value of the resulting regression will increase. This procedure was applied for each probe. The differences in the P-values (z-values) obtained in this way, are shown in table 5A. The data show that the probes differ widely in their contribution to the age effect. More than half are not involved and the deviation index calculated from these 10 probes does not correlate with age (P = 0,798). However the deviation index calculated from the 8 probes which do contribute, correlates strongly with age (P = 0,00027) with the 6 most influential probes being possibly even more strongly correlated (P = 0,00024). The reason for this probe specificity in aging remains a mystery, but the z-values (Table 5A) of the 8 probes providing the greatest contribution correlate significantly (P = 0,0086) with the slopes of the regression lines relating proteasome expression level and "log obs/exp" (Table 2). Therefore it appears that this aging phenomenon might be connected to the degree of upregulation and downregulation in the expression of these genes.

**Table 5:** Effect of leaving out the data from one probe on the regression between age and deviation index. The change in P-value has been transformed into a change in z-value. Positive z-values indicate a contribution of the probe to the regression. Regression was calculated for "2^nd^-log obs/exp" (section A) and for "transformed 2^nd^-log obs/exp" (section B).

A										
	omitted probe	A7b	B7c	B7b	B5	A7a	A1	A2a	A2b	A3a
	change in z-value	-0,2436	-0,2252	-0,1506	-0,1307	-0,0344	-0,0045	0,0000	0,0000	0,0000
	omitted probe	A3b	A5	B6	A4	A6	B7d	B2	B7a	B3
	change In z-value	0,0000	0,0033	0,0233	0,0335	0,0472	0,0587	0,0832	0,6345	1,3107
B										
	omitted probe	B5	A3a	A3b	A7b	B7c	A2b	B7d	B7b	A1
	change in z-value	-0,2393	-0,2149	-0,2014	-0,1172	-0,1140	-0,1109	-0,0852	-0,0171	-0,0092
	omitted probe	A7a	A5	A2a	A6	A4	B6	B2	B7a	B3
	change In z-value	0,0065	0,0191	0,0398	0,0977	0,1219	0,1498	0,2637	0,2975	0,5399

To determine to what extent the age factors contribute to the variation in "2^nd^-log obs/exp", regression lines (calculated between age and the "2^nd ^– log obs/exp" for each probe – data not shown) were used to eliminate the effects of age from the "2^nd^-log-obs/exp" in the same way in which the influence of proteasome expression level was previously removed. This procedure therefore results in removal of the effects of age for the type II and type III probes described above as can be seen by, for example, the change in the P-value of the regression between age and the mean "2^nd^-log obs/exp" of type II probes from P = 0,002 to P = 0,996.

However the reduction in the variance of "2^nd^-log obs/exp" is only 4,6% and the regression between age and deviation index is almost unaffected: P = 0,00049 instead of P = 0,00027. It would therefore appear that two, largely independent, age effects exist, one connected with the absolute expression of a gene in relation to age (age-factor 1) and the other with the residual variation possibly connected with variation between individuals or with the interrelation of expression of the different genes (age-factor 2).

**Figure 8:**
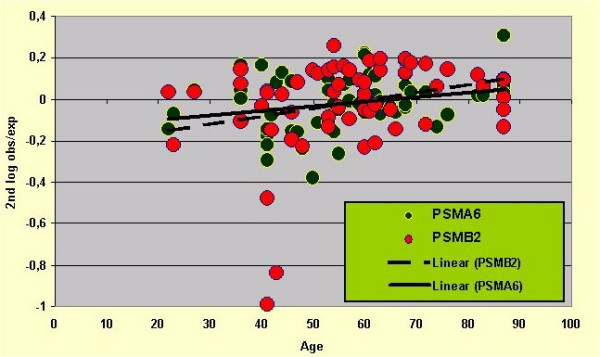
The regressions between age and 2nd log obs/exp for two genes suggest the presence of the two age effects. Age effect 1: 2nd log obs/exp goes up with age. Age effect 2: degree of scattering of data points around the regression lines decreases with age.

To visualize these two age effects, the relation between age and 2^nd ^log obs/exp for two genes (PSMA6 and PSMB2) is shown in Figure 8. These two genes were combined since they happen to have comparable regression lines. The first age effect is seen in the slope of the regression lines, 2^nd ^log obs/exp going up with age. The second age effect is seen in the scattering of the data points around these lines, suggesting a decrease in scattering with age.

### Aging and gender

The wide variation between individual probes in the mean and standard deviation of the "2^nd^-log obs/exp" (Fig. 3) might underlie their differing contributions to the regression with age. However, transformation of all these probe distributions to ones with the same mean: "0" and the same standard deviation: "1", (data not shown) and then use of this "transformed-2^nd^-log obs/exp" data to recalculate the regression between age and deviation index did not alter the P-values (P = 0,0112 instead of P = 0,0117). However, omission of individual probes from the regression with age analysis in order to determine their contribution, resulted in a slightly different pattern (Table 5B) in which stronger regressions with age were observed with the most pronounced being those of the 6 most contributing probes (P = 1,39 × 10^-5^).

**Figure 9:**
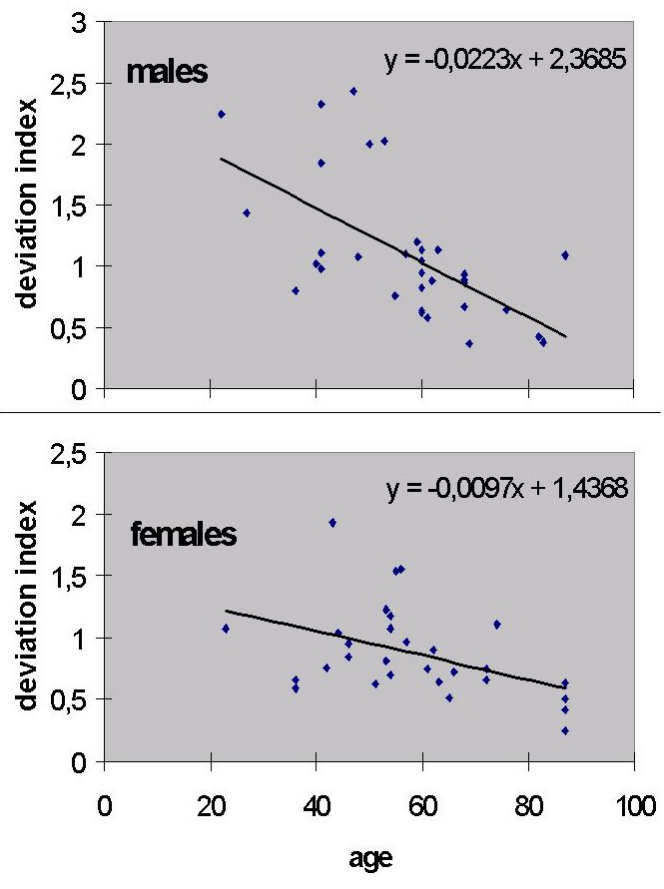
Difference between males and females in the age related deviation index (P < 0,0001, ANCOVA).

This highly significant age effect allowed us to assess whether a gender-based difference in aging was demonstrable. The data presented in Figure 9 show that this was indeed the case, with the regression lines for both females (P = 0,0184) and males (P = 0,0002) being significant. After ANCOVA analysis, (performed in order to determine whether these regression lines differed for gender), a highly significant interaction between gender and age was observed (P < 0,0001). The P-values for the differences in intercepts and slopes were 0,022 and 0,059 respectively, demonstrating that males and females do differ in aging processes.

This finding, (if confirmed), might well alter our current views of aging.

### Tissues and remaining factors

Heterogeneity between tissues might contribute to the variation in "2^nd^-log obs/exp". If the libraries are grouped into 5 classes by k-means clustering, there appears to be a tendency for clustering in the same class for some of the tissues (Table 6).

**Table 6:** K-means clustering of "2^nd^-log obs/exp" into 5 classes reveals a tendency for tissue specificity

class 1	class 2	class 3	class 4	class 5
adrenal 1	adrenal 2	adrenal 3	brain 4	cervix 3
bladder 1	bladder 2	adrenal 4	ovary 3	colon 3
bowel 2	bowel 1	bowel 3	prostate 1	diaphr
brain 6	brain 1	brain 3	prostate 2	fallop t 4
breast	brain 2	brain 5	sem ves 1	heart 3
colon 2	brain 7	cervix 2		lung 2
epidyd	brain 8	colon 1		lung 3
fallop t 2	cervix 1	esoph 1		muscle 2
lung 4	esoph 3	esoph 2		pericard
ovary 1	fallop t 1	heart 1		saliv gl 1
ovary 5	fallop t 3	heart 4		saliv gl 2
stomach 1	gallbl	heart 6		saliv gl 3
stomach 4	heart 2	kicney 3		saliv gl 4
testis 1	pancre 1	kidney 4		stomach 2
	prostate 3	kidney 5		stomach 3
	prostate 4	liver 1		testis 2
	prostate 5	liver 3		uterus 5
	uterus 1	liver 5		vagina
	uterus 2	lung 1		
		muscle 1		
		ovary 2		
		placenta		
		sem ves 2		
		sem ves 3		
		testis 3		
		uterus 3		

**Figure 10:**
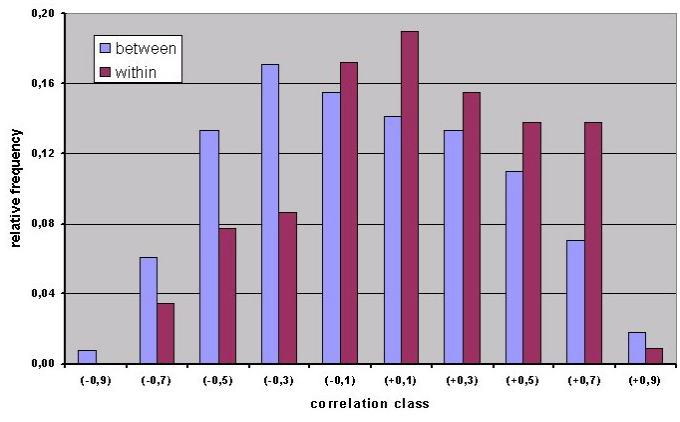
Histograms of the frequencies of correlation coefficients from pairs of libraries derived from different tissues (between) and from pairs of libraries derived from similar tissues (within). The means of the distributions are respectively: -0,011 and 0,133 and the correlations differ significantly (Mann-Whitney: P = 0,00027).

To determine whether the transcription profiles from libraries obtained from the same tissue type are indeed more similar to each other than profiles originating from different tissues, the correlation coefficients of all pairs of libraries were divided into two groups. In one group, libraries *within *tissue types were compared whilst in the other, comparisons of libraries *between *tissue types were made. Histograms of these correlation coefficients are shown in Figure 10. Relatively, more positive correlations were found for the intra-tissue group, with the difference between the two groups being significant (Mann Whitney: P = 0,00027). This means that there is indeed a tendency for similar tissues to exhibit similar expression profiles. Additional evidence in support of this conclusion was obtained by ANOVA analysis of 18 tissues, each represented by 3 libraries. The results revealed a significant interaction between tissue and probe-set (P = 1,99 × 10^-5^) and were therefore in agreement with the effect of differentiation on the transcription profile.

Another ANOVA was performed using the same 18 tissues and replicates for the 6 probes used to generate the age-related deviation index. In this case there was no suggestion of heterogeneity between tissues (P = 0,133), indicating that this index and the observed effects of age and gender are not influenced by tissue heterogeneity.

However, different gene probes from the same probe set display another type of heterogeneity. Table 2 shows that for B7d a positive correlation exists between "log obs/exp" and proteasome expression level (+0,398), whilst for B7c this correlation is negative (-0,302); for both probes, the correlations are highly significant: P = 0,00021 and P = 0,0059 respectively. A similar situation is seen if one compares the pair of probes for A3. Thus it could be that the different gene probes of a set sometimes identify different RNAs.

An ANOVA for B7c and B7d (using18 tissues with 3 replications, as above) but this time using the '2^nd^-log obs/exp" data reveals that the heterogeneity of these probes relates to different tissues (P = 3,5 × 10^-5^). This was also true for the two A3 probes (P = 0,00036) with the A7 probes showing the same tendency (P = 0,087). However, no such effect was found for the two probes of A2 (P = 0,58). Such heterogeneity could be due to alternative splicing products [16] although it is hard to imagine that the structure and function of the proteasome would vary between different tissues.

### Disorder in transcription

It is possible that still another factor may be related to the variation in "log obs/exp" since 8 libraries with outliers were omitted from the analysis. Because one of these libraries (pancreas 2) simultaneously displayed 5 outlying "log-obs/exp", it would appear that the origin of outlying values is associated with the tissue rather than with 'noise' or experimental error. Perhaps normal tissues can exhibit general disturbances in transcription profile patterns comparable to those previously described for SAGE data [2].

### Postscript 1: SAGE and micro array complement each other

Since the first observations on epigenetic hereditary transcription profiles were made using SAGE data and the present ones with micro arrays one wonders to what extent these data are interchangeable.

The mean transcriptional profile of the proteasomal genes obtained with SAGE data was found to differ from that obtained with micro array. This is not so surprising since the expression data are obtained in very different ways. That SAGE and micro array data do complement becomes visible after calculation of log obs/exp. Both the SAGE database and the micro array database have 8 libraries derived from normal brain. After calculation of log obs/exp for each proteasomal gene, significant correlation of these two deviation profiles was observed (R = 0,75 P = 0,0051).

### Postscript 2: meaning of the findings

A number of puzzling observations have emerged from this study. For example, the finding, (using a limited number of libraries), that the transcription of only 12 genes demonstrates specific patterns in the deviations of the mean transcription profile in relation to cellular aging, gender and differentiation. This is intriguing since these genes are not themselves involved in the regulation of other genes. These patterns are even present in the residual values after correcting "log obs/exp" for proteasome expression level and for age. It would thus appear that a fundamental characteristic in gene expression may have been uncovered. The factor involved could be epigenetic inheritance since epigenetic variation will be transmitted in the cell lineage and can undergo some change during transmission [2].

Similarly, the apparent wide variation in the transcription profile of the proteasomal genes is puzzling because a corresponding biological role cannot be assigned and also because it is hard to imagine how the structure of the proteasome organelle might be affected by these differences in profile. Rather, the observed differences in proteasomal transcription profiles would seem to reflect a tolerability of the cell to such variation.

There does not appear to be any frame of reference within our current knowledge-base by which these unusual findings can be interpreted. The following comments (which have been added as a post-script, since they represent at best a theoretical pre-scientific pondering) have therefore been included in an attempt to provide a possible explanation for this enigma.

It has been repeatedly argued that the study of biology requires a new approach which incorporates a shift in thinking from reductionism towards a more holistic perspective [17]. In this way, the cell would be viewed, not as an aggregate of molecules, but rather as a complex integrated system. It is to be expected that we will eventually be confronted with phenomena that can only be explained from this viewpoint. This could in fact be the case now.

One of the characteristics of a complex system is that oscillations occur. Presumably an oscillation cannot be in homeostasis if left to itself outside of a regulatory context. Due to intrinsic noise it will either decrease or increase in amplitude ending ultimately in extinction or crisis, respectively.

Within a living cell, however, an oscillation is regulated and not left to itself. An oscillation hierarchy probably exists in which higher-order oscillations determine the fate of lower ranking oscillations. A good example of a higher-order oscillation might be the circadian rhythm, since this rhythm will interfere with many other oscillations within the cell so as to promote order.

However, one might question whether a system of interrelated oscillations can really be in a permanent state of homeostasis, since a complex system of oscillations may also be subject to the same fate as that of a single oscillation. In that case it would be advantageous for a cell if its fate consisted of a very gradual decline of oscillations accompanied by a correspondingly gradual decline in cellular function. Such a gradual decline in function is known to occur during aging. Aging could then act as the highest-ranking oscillation, in which a high deflection present at birth gradually declines over time until function is too severely compromised and death occurs. Cellular aging would therefore be the ultimate organizing principle, necessary for long-term maintenance of cellular function. The mechanism by which this might come about could be a progressive epigenetic hereditary alteration in chromatin organization.

This theoretical view allows for the possibility of both chance events with no or negligible biological consequences and for interference in the process of aging by damaging agents. This theory might also help in finally resolving the longstanding dispute about the cause of aging. This controversy, which has recently flared again in relation to the use and applicability of mutant mouse strains in aging research [18-21], is based on two opposing theories of aging. In one view aging is thought to result from the gradual accumulation of damage, whilst in the other, the aging process is considered to be an intrinsic developmental program.

The foregoing has however suggested a third possibility, i.e. that aging results as a consequence of fundamental laws associated with complex systems. From this alternative viewpoint, the concept of 'aging as a program' does not need to be invoked, rather aging is considered to be as self-explanatory as water running downhill, although the understanding at the molecular level remains a major task. Thus this very process of aging could, in principle, function as a way to prevent malfunctioning and the accumulation of damage.

The analytical method which has led to this novel view of aging can also be applied experimentally to solve the key question in aging: i.e. "which changes associated with the aging process are causative and which are merely secondary consequences?"[22]. This method of measuring the deviation index in transcription profiles, (if validated by further research), could be used to discriminate between aging being due to a decrease in deviation index (as described in this paper), or due to the occurrence of pathological events or genotoxic stress, the latter being characterized by an increase in deviation index as found previously for carcinogenesis [2].

Another consequence of this theoretical approach is that aging could be seen as a protective mechanism that might accelerate in response to mutational damage, rather than the view (gaining currently in popularity) that aging is a result of the accumulation of mutational damage [23-25].

In addition, the method described in this paper can identify tissues that differ widely in hereditary transcription profiles of the proteasome genes and which might thus be helpful in elucidating the nature of the underlying epigenetic hereditary structure.

As pointed out by the 2^nd ^reviewer the age related decrease in deviation of transcriptional profiles reflects the increase in regular behaviour as a consequence of chaotic dynamics, described for human physiology [26]. This strengthens the view that the explanation of the observed phenomena has to be sought in the nonlinear dynamics of complex systems.

## Materials and methods

The 14 genes of the 20S proteasome are : PSMA1, PSMA2, PSMA3, PSMA4, PSMA5, PSMA6, PSMA7, PSMB1, PSMB2, PSMB3m PSMB4, PSMB5, PSMB6, PSMB7. When more than 1 probe is available for a particular gene (e.g. PSMA2) the transcript abundances for the two probes have been recorded as for PSMA2a and PSMA2b.

For this analysis sheet 5 of "Additional File 2" obtained from the database of normal tissues (GSE 2193) was used [27]. This sheet provides the abundance compared to genomic DNA. From this sheet abundances were calculated by using the data as the power of 2. The database provides no information on the expression of PSMB1 and PSMB4. Two probes for the expression of PSMA2, PSMA3 and PSMA7 and four probes for the expression of PSMB7 are available. The data for the separate probes within each probe-set were analysed independently. Tissues that might possibly express the immunoproteasome were omitted leaving 90 tissues for analysis. Missing values were estimated using the principal component analysis (PCA) from XLSTAT.

The mean relative transcription profile was obtained by determining the contribution of each probe (as a percentage) to the total of all abundances. Expected abundances were calculated by multiplying the relative contribution of each probe with the proteasome expression level (total of abundances for a specific library). Using the observed and expected abundances "log obs/exp" was calculated. The results, in Additional file 1, show the deviations from the mean transcription profile; positive values represent relative over-expression and negative values relative under-expression. The age of the donor was known for 68 of the 90 libraries. For the ANOVA of 18 tissues with 3 replicates, excess in available replicates was reduced by chance. All calculations were performed with XLSTAT.

## Competing interests

The author(s) declare that they have no competing interests.

## Reviewers'comments

### Reviewer's report 1

Dr. Timothy E. Reddy, nominated by Dr. Charles DeLisi, Boston University, Boston, United States

### Final comment after examination of the author responses

Upon clarification and revision, I am able to take much more positively to the manuscript. I greatly appreciate the responses to the critiques made, and have no others to make. As such, I now support the publication of the manuscript.

### Original comments

I have major problems with the manuscript. First, the materials and methods throughout the paper are insufficiently described; and much of the statistical analysis is either insufficiently described or inadequately presented. These problems make it difficult to assess the scientific merit of the conclusions. Second, many of the conclusions in the paper are unjustified. However, it is not clear how much of this is due to difficulty understanding the methods. The paper lacks clear directions, and I am unsure what the main scientific conclusions of the paper are. Finally, the paper concludes with a lengthy, unrelated, and unjustified discussion attributing explaining aging as "self-explanatory" and a unavoidable consequence of otherwise unnamed "fundamental laws".

#### Author response

*The following will make it clear to what extent these statements hold true*.

In particular:

There are insufficient details about the set of 14 genes used for study. How are the 14 proteosomal genes selected? Are they part of the core 20S proteosome complex, or part of a regulatory complex? It would be very useful to at least list the 14 genes used for study.

#### Author response

*As this paper is a sequel to an earlier paper on "Epigenetic Hereditary Transcription Profiles" a reference was made to this paper both for the rationale of the choice of the 14 genes of the 20S proteasome and for the use of the "mean transcription profile" as tool. The information on these issues provided in the present paper has been improved in order to improve the readability and the genes have been listed in "Materials and Methods"*.

There is also insufficient details about the microarray data used in the study. Here, an explicit description of the microarray data, the number of and types of samples in the database, and how "absolute transcript abundances" are calculated is necessary to understand the rest of the paper.

#### Author response

*Since the database is not mine but has been derived from the literature the full details of the data have to be found in the cited reference. In "Materials and Methods" more information has been provided on the type of the data and the calculation of the abundances*.

Table 1B and Table 4 are not useful to the reader, and would be better included as supplementary info. Instead, a summary of the data that is easy for the reader to interpret would be more appropriate.

#### Author response

*Since *Table 1B*contains the basic material for all calculations it was left in place. Since this does not hold for *Table 4, *this table has now been included as an additional file*.

PCA is used to analyze the data, but this is not fully explained. What are the dimensions for the PCA? What are the most important factors (eigenvectors)? The author refers to F2, F3, F4, and F9. What are the major components of these vectors? As it stands, the results from the PCA are insufficiently described to be useful to the reader.

#### Author response

*The PCA was used solely to assess whether the two quantitative variables of the database (proteasome expression level and donor age) could affect the proteasome expression profile. Since the answer is a clear yes the remainder of the analysis aims to describe this result further. For a better understanding of this point the text was improved*.

I feel the first paragraph under "Expression level" is completely unsubstantiated. Why does considerable variation in individual tissues evidence against epigenetic structures? It seems completely reasonable to me that different epigenetic structures in different tissues could contribute to expression variability. Moreover, the assessment of variability is based on 4 samples from 4 different individuals, and are far from conclusive.

#### Author response

*Indeed the data do not allow deciding what part of the variation is due to gene regulation by transcription factors and what to epigenetic structures. The text has been modified accordingly*.

*To me it is not clear why the assessment that there can be variability in proteasome expression level within one tissue type for different individuals would not be valid*.

In the next paragraph, it is unclear what can be gleaned by plotting the expression level of a gene against the log(obs/exp) value. What is the difference between the expression level of a gene and the "obs" factor in the log formulation? They seem to be identical to me (the abundance of a gene in an array), and if they are not, perhaps a better explanation of the difference would be useful. Either way, it is unclear why "widely different slopes" would imply that expression level affects the transcriptional profile. Looking at the slopes presented in Table 3, the slopes all appear to be on the same order of magnitude, and do not seem to follow any intuitive definition of "widely different". Is there a correlation between expression level and the slope? Are higher expression levels correlated with steeper slopes? What are the correlation coefficients for the regressions? As for the identification of outliers, it would be better to choose outliers based in a more rigorous way.

#### Author response

*Here a basic misunderstanding, essential for understanding the paper, occurred. The expression level of a tissue is the sum of the abundances of all probes, as such stated in the 2*^nd^*paragraph of the Results and implicitly also in Materials and Methods. The slopes are widely different. Some probes even have negative slopes, which means that tissues with a high proteasome expression level have relatively less transcripts for these probes than tissues with a lower proteasome expression level, in other words: the transcriptional profile is different for different proteasome expression levels. To avoid such misunderstanding the term "expression level" has been replaced by "proteasome expression level" throughout the text. This term is also introduced in Materials and Methods*.

The regression results are called into question by results for the two probes for the B7 gene. As shown in Table 3, there is a negative correlation with probe B7c, but a positive correlation with probe B7d. This issue needs to be addressed.

and

The regressions are repeated by correlating expression level with transcript abundances. However, it is stated earlier that the expression level is the transcript abundance, and it is unclear what the difference between the two values is. Moreover, the data for the regressions is not even loosely described, and therefore any conclusions about the proteasomal genes based on the regressions are unsubstantiated.

#### Author response

*The previous author response shows that the misunderstanding caused the impression that these issues were not addressed although they were addressed*.

What does it mean to "upgrade the expression level of the proteosomal genes". I have never heard the term, and there is no explanation of it in the text.

#### Author response

*The term "to upgrade the expression level of the proteasomal genes" had been used as a synonym for upregulation, Since, strictly speaking, it is not known whether the differences between tissues in proteasome expression level are due to regulation the use of both terms is now avoided*.

At this point, I can no longer follow the analysis. What does it mean to calculate the transcript abundance of a gene at an expression level of 50 or of 200? The difference between expression level and transcript abundance remains unclear. The induction measure is based on two points, and no indication of statistical significance is provided. What is the justification for the 4 classes of induction? How are the boundaries between the classes chosen?

#### Author response

*As stated above the misunderstanding is connected with missing the difference between (proteasome) expression level and the (probe) transcript abundance. The text has been adapted to avoid this misunderstanding*.

*The division into 4 classes is just for a better grasping the differences in expression, therefore the boundaries are rather arbitrary. This has now been stated in the text*.

### Removal of the influence of expression level

How is "26.1%" calculated?

#### Author response

1-(0,2629/0,3558) = 0,261 has been added to the text

In figure 2, some measure of variance should be shown, as error bars, on the plots. As it stands, it is impossible to assess the statistical significance of differences between means, and to make any substantiated conclusions based on those differences.

#### Author response

*Standard errors have been added*.

The results from the k-means approach seem contradictory to an earlier statement in the paper. Here, it is stated that age is correlated with an under expression of proteasomal genes. However, earlier in results, it is stated that age does not significantly correlated with expression. Perhaps the discrepancy can be resolved by regressing age with individual gene probes, or with a combined set of gene probes? It should also be mentioned that the 4 probes only correspond to 2 genes.

#### Author response

*It was stated that age did not correlate with proteasome expression level. The k-means clustering however did not sort the data to proteasome expression level but to log observed/expected, thus to degree of deviation of expected abundances. The text has been made more explicit*.

### Age Factor 1 and Age Factor 2

I would suggest more useful section headings.

The section headings were changed into:

*Age factor 1 (age related variation in mean transcript abundances)*

*Age-factor 2 (age related variation in residual values)*

*Moreover to improve the understanding of the two age factors *figure 8*was added*.

The division of ages into three groups seems arbitrary. How were the age boundaries selected, and are the results sensitive to the boundaries chosen? What if more or fewer groups are used? Perhaps a sliding window approach would be more convincing.

#### Author response

*The division into three age groups was made such that the three groups have about the same size and have a specific boundary. This could be called arbitrary and possibly a sliding window approach could avoid this. The latter approach is however not available in XLSTAT. The convincing part of the section is however in the significant regressions found between age and transcript abundance for some probes (= age factor 1)*.

How is the dissimilarity threshold chosen for group determination in hierarchical clustering? It is disturbing that different probes for the same genes are clustered into different groups.

#### Author response

*The dissimilarity threshold was chosen automatically as AHC was set for 3 classes. The problem that different probes of the same genes behave differently was discussed in the section on "Tissues and remaining factors"*.

In figure 5, again, there is no indication of variation for the different bars, and no indication of statistically significant differences.

#### Author response

*Standard errors have been provided*.

Figures 6 and 7: Please report correlation coefficients for the regressions

#### Author response

*Correlation coefficients have been provided*.

For the leave one out regression, it would be more satisfying to see what the change in correlation coefficient is with the re-addition of each probe.

#### Author response

*Possibly. The change in correlation coefficient by leaving one out regression was checked and was found to lead to an identical result. Therefore things were left as they are*.

### Tissues and remaining factors

How is the tendency for similar tissues to group together measured? As I look at table 6, it seems that almost every tissue is found in more than one cluster. Only Kidney, Liver, and Salivary Glands all group into the same cluster, and many tissues are found in 3 or more different clusters.

#### Author response

*As mentioned, the grouping together was measured by k-means clustering. The table only suggests that there is some grouping together at least for some tissues. This last remark has been added to the text*.

For the inter vs. intra tissue correlations (Figure 10), it would be useful to report the mean of the distributions, and perhaps plot them on the same axes so it is easy for the reader to assess differences between the distributions. Also, the chi-squared test sums over a set of possible outcomes. What are the outcomes used here? Perhaps a T-test (if the distributions are normal), a ks-test, or a Mann-Whitney U test is more appropriate?

#### Author response

*The means of the distributions have been added and the histograms were plotted on the same axes. Mann-Whitney has been applied instead of chi-square*.

Simons explains the opposite direction correlations in figure 2 as the result of different probes measuring different RNAs, but there is no justification of the conclusion. Alternative explanations such as natural variance in the microarrays are not mentioned. Moreover, without any indication of the correlation coefficient, the reported p-values have little meaning.

#### Author response

*Both correlation coefficients have been added and their significance. The explanation of "natural variance" escapes me but our explanation has been mitigated*.

##### Postscript

The postscript begins by suggesting a "fundamental factor in gene expression may have been uncovered." The discussion does not, however, name the factor. Moreover, the idea that the expression of a set of genes is different in different cell lines, in different tissue types, and in different genders is not surprising. In fact, the contrary, that the 12 genes showed the same expression level across tissues and time, would be a much more intriguing result.

#### Author response

*What has been observed is not just some variation in expression levels of some genes but systematic patterns in the deviations from the mean transcription profile in relation to age and differentiation. These patterns are even still present in the residual values after correcting "log obs/exp" for proteasome expression level and for age and these patterns do not seem to be related to gene function. The factor could be epigenetic inheritance since epigenetic variation will be transmitted in the cell lineage and can undergo some change during transmission. This remark has been added to the text*.

Simons goes on to suggest that there is no way to reasonable explain the findings. First, it is unclear the specific findings to which Simons refers. However, numerous simple explanations are possible: It is more than likely that the rate of protein degradation is different in various cell types, and that increasing or decreasing the rate of protein turnover may have important consequences. Moreover, transcription is only a first step in gene regulation, and there is no attempt to measure the effect on ultimate protein levels. It is entirely possible that posttranscriptional modifications buffer differences in transcriptional profiles.

#### Author response

*The specific findings and their possible explanation have been dealt with in the response to the previous comment. The role of posttranscriptional modifications is of course well known but could only explain why differences in transcription do not necessarily lead to different proteasomes and are not an explanation for the observed phenomenon*.

The discussion then moves into a discussion about a hierarchy of oscillatory rhythms, with aging being the highest order "rhythm". Although the discussion is stated as hypothetical, doing so does not justify making grand and unrelated conclusions based on a limited sample of 12 genes. The proposed hierarchy of rhythms is entirely disjoint from the remainder of the paper, and bears little if any relation to the results presented. Furthermore, the suggestion that aging is the result of a fundamental law of complex systems is entirely unsubstantiated, and not even remotely suggested by the work presented. The analogy to water running downhill is entirely inappropriate: water running downhill is attributed to the force of gravity, interactions between water molecules, and these effects are studied by a large body of experimental physics: if water running downhill seems "self-explanatory", it is only because centuries of scientists have studied the topic. To attribute aging to an unnamed and entirely mysterious "fundamental law" is entirely unscientific, and should not be published in a scientific journal.

#### Author response

*As stated in the postscript this part of the discussion is hypothetical and even "prescientific". So there are no grand and unrelated conclusions, only a new phenomenon that needs to be explained. As stated also there appears to be no frame of reference that can explain the results, therefore one has to search for something different. It is entirely justified to propose that the phenomenon could be connected with the cell as a complex system. I agree that this is not yet scientific but it can be called "pre-scientific" and in line with the unfolding of science over centuries*.

*The analogy to water running downhill seems appropriate to me in sofar as it refers to the "normality" of all processes of deterioration that affect our car, house etc, understanding at the molecular level is of course a different thing. This has been added to the text*.

### Author response to the introductory comments

#### Introductory comments

I have major problems with the manuscript. First, the materials and methods throughout the paper are insufficiently described; and much of the statistical analysis is either insufficiently described or inadequately presented. These problems make it difficult to assess the scientific merit of the conclusions. Second, many of the conclusions in the paper are unjustified. However, it is not clear how much of this is due to difficulty understanding the methods. The paper lacks clear directions, and I am unsure what the main scientific conclusions of the paper are. Finally, the paper concludes with a lengthy, unrelated, and unjustified discussion attributing explaining aging as "self-explanatory" and a unavoidable consequence of otherwise unnamed "fundamental laws".

#### Author response

*To me it seems that the basic problem for the reviewer is that the core of the research was not grasped. Partially this might be due to the subject itself as it is a new phenomenon and the paper is just a search for its approach. Especially if the foregoing paper on "epigenetic hereditary transcription profiles" was missed difficulty in understanding could arise. As far as possible all mentioned problems on materials and methods throughout the paper have been dealt with and certainly this will contribute to a better understanding*.

*The reviewer also missed the distinctions made between hypotheses, indications and conclusions. Practically the only conclusion is that a new not understood phenomenon is at hand with respect to transcription during aging and differentiation that needs to be studied. In the "postscript" an answer is sought in the cell as a complex system. Since this can hardly be called scientific it has been named ""pre-scientific pondering", which to me is allowed in this particular case*.

*Therefore, despite the negative judgment of the reviewer, only publication of the paper can make the phenomenon public and accessible for further research, which to me appears necessary*.

### Reviewer's report 2

*Dr. Nathan Bowen, nominated by Dr. I. King Jordan, School of Biology, Georgia Institute of Technology, Atlanta, United States*.

In this manuscript, JWIM Simons complements a previous analysis of the global transcriptional profiles of proteasomal genes. In the first study, SAGE data was used, whereas in this study, expression microarray data is used. As many issues to the motivation and examination of this kind of data have been discussed in the previous review, I will not dwell on them here.

In this study, the sample group is large (90) and many life history traits are known for the samples. One major finding is, that for proteosomal genes, the deviation from an average transcriptional profile decreases as age increases. There is also a gender specific component to the deviation. In this study, gene expression measurements were collected from normal tissues. After consideration of this analysis and results, it seems to me that this finding is in direct support of previous observations made by Bruce West and colleagues (see below, this quote should be checked for accuracy as I was not able to find an original copy of the article by West et al. and took this from an online commentary):

"The conventional wisdom in medicine holds that disease and aging arise from stress on an otherwise orderly and machinelike system – that the stress decreases order by provoking erratic responses or by upsetting the body's normal periodic rhythms. In the past five years or so, we and our colleagues have discovered that the heart and other physiological systems may behave most erratically when they are young and healthy. Counterintuitively, increasingly regular behavior sometimes accompanies aging and disease. Irregularity and unpredictability, then, are important features of health. On the other hand, decreased variability and accentuated periodicities are associated with disease. Motivated by these ideas, we and other physiologists have looked for periodic behavior that might indicate developing sickness (especially diseases of the heart). In addition, we have begun to analyze the flexibility and strength of irregular fractal structures and the adaptability and robustness of systems that exhibit apparently chaotic behavior."

Chaos and Fractals in Human Physiology, by Ary L. Goldberger, David R. Rigney and Bruce J. West, Scientific American February 1990, pp.35–41

It may be that the methods described herein by Simons are indeed a direct measure of aging and/or disease. The decrease in deviation of transcriptional profiles observed by Simons may reflect an increase in regular behavior as described by West. In my opinion, this is a very interesting finding.

This manuscript emphasizes the strengths of global analyses and the often-unforeseen trends that emerge from rigorous examination of genomic data.

Minor critiques:

-The author should compare/contrast the findings from the SAGE Analysis to this analysis in a bit more detail, expanding on the last sentence of the discussion.

#### Author response

*Two comparisons between SAGE and micro array data are made in postscript 1*.

-Perhaps West et al. provides a frame of reference for further interpretation by the author in the discussion.

#### Author response

*This reference is very welcome and has been included in the list*.

-The postscript should be formally incorporated into the discussion section.

#### Author response

*The postscript has been incorporated into the discussion section as a subheading*.

-Could the author please expand and clarify the statement:

*"Since considerable variation can be seen in individual tissues, e.g. adrenals (75–174) and salivary glands (63–124), it seems that this variation in expression level is largely related to gene-regulation and not to epigenetic structures*.

I am a bit unclear as to the conclusion. Do epigenetic structures restrict variation?

#### Author response

*The sentence has been corrected since the data do not justify the statement*.

### Reviewer's report 3

Dr. Martijn Huynen, Center for Molecular and Biomolecular Informatics, University Nijmegen Medical Centre, The Netherlands.

The paper by Johannes Simons analyzes the level of gene expression of a number of proteasome genes across a number of tissues and people in order to study natural variations in gene expression in a set of fuctionally related genes. The ultimate goal of the project is to shed light on epigenetic factors.

I do have significant issues with this manuscript, both technical and conceptual. In general I seriously doubt whether one can draw biologically meaningful conclusions just from analyzing a single dataset like that. Of course one will observe variation, and of course on will find that the total level of gene expression correlates with that of the genes that make up the sample (page 6, Table 2), but I do not find this constitutes "enough" novelty or biological insight to warrant publication. The main conclusion, that the variation in gene expression would become smaller with age is interesting, but I am not convinced that the results are reproducible, specifically given issues like the different behaviour of probes from the same genes in the data.

#### Author response

*Whether the conclusions are meaningful depends on to what extent this dataset of a small number of genes is representative for all genes, it might be and this has also been stated in the text*.

*It seems that this summing up of the novelty does not result from an objective attempt to describe the main findings of the paper*.

##### Specific comments

Why were proteasome genes chosen for this research question? And why this specific set?

#### Author response

*As stated in the introduction (and also in the foregoing paper) the set of proteasome genes was arbitrarily chosen as model system for a group of functionally related genes. The choice could have been any other group of functionally related genes. The proteasome genes appeared suitable as the genes are all located on different chromosomes and regulated independently*.

Why include autopsy data, in some cases 71 hours post-mortem? Would that not give rise to biologically not relevant effects?

#### Author response

*Whether the post mortem data had to be included was investigated *(table 4). *Since no indication for a difference with biopsies derived from surgery was found they were included in the analysis*.

A first PCA factor that explains 31% is in my experience quite significant, and I would be highly intrigued to know the interpretation of this factor.

#### Author response

*This particular factor was found to correlate significantly with donor age and to have no correlation with proteasome expression level*.

The tables deserve more extensive legends: I do not know what "expr. Level" means?

#### Author response

*Legends have been improved*.

To state that individual genes are differently affected by the total amount of variation in transcription we need statistics.

#### Author response

*In *table 2*is shown that the expression measured with 4 probes correlated negative and significantly with proteasome expression level whereas another group of probes correlated significantly positive. The differences between the probes therefore have to be significant*.

How to explain that different probes from the same gene fall in the three different classes A, C and D? Is this biology? Or is this experimental noise? And if this is experimental noise how does it affect the conclusions obtained?

#### Author response

*That different probes from one probe can behave very differently was a surprise. The cause has to be sought in biology since this probe heterogeneity was connected with tissue heterogeneity (section" tissues and remaining factors")*.

Why is the considerable variation observed between the tissues not due epigenetic factors (page 5)?

#### Author response

*Variation can be due to either regulation by transcription factors or by epigenetic factors. The text has been improved*.

Printing such a large table as Table 2 is a waste op paper.

#### Author response

*Yes but is seems compatible with open access*.

I do not understand the procedure on page 7, with clustering followed by statistical tests (ch-square or wilcoxon). Is there a more direct way of doing this analysis?

#### Author response

*That procedure was to assess whether the data could be informative on age, gender and biopsy. No doubt there will be better ways to perform the whole analysis. With the results obtained we will now be able to develop a more transparent methodology*.

Do I understand correctly that the aging effect is only significant for the middle-age to aged comparison? how should we interpret that ? would one not expect to seem the most dramatic difference between the youngest and the oldest cohort?

#### Author response

*Could be but It is equally possible that age related changes show up as a humped curve, only further research will tell*.

The clustering on page 8 again shows the inconsistent behaviour of the different probes per gene. In the presence of such inconsistencies I doubt the biological relevance of the results.

#### Author response

*The different behaviour of probes from one probe set might be contrary to expectation however it is observed and seems to be connected with differentiation. Whether this is inconsistent with belief or with facts remains to be seen*.

## Supplementary Material

Additional file 1 Log obs/exp of transcript abundances from all 90 libraries for all 18 probes. First treatment of raw data. Click here for file

Additional file 2 Values for "log obs/exp" after removal of the variation due to proteasome expression level (= "2^nd ^log obs/exp"). Second treatment of the data. Click here for file
