# Supplementary material for: Epigenetic hereditary transcription profiles II, aging revisited
Source: Biol Direct. 2007 Dec 28;2:39. doi: 10.1186/1745-6150-2-39 (PMC2265679; doi:10.1186/1745-6150-2-39)
Supplement: Additional file 1 — Log obs/exp of transcript abundances from all 90 libraries for all 18 probes. First treatment of raw data. [file 1745-6150-2-39-S1.doc]

Additional file 1. Log obs/exp of transcript abundances from all 90 libraries for all 18 probes.

|  | log obs/exp | |  |  |  |  |  |  |  |  |  |  |  |  |  |  |  |  |
| --- | --- | --- | --- | --- | --- | --- | --- | --- | --- | --- | --- | --- | --- | --- | --- | --- | --- | --- |
| tissue | PSMA1 | PSMA2a | PSMA2b | PSMA3a | PSMA3b | PSMA4 | PSMA5 | PSMA6 | PSMA7a | PSMA7b | PSMB2 | PSMB3 | PSMB5 | PSMB6 | PSMB7a | PSMB7b | PSMB7c | PSMB7d |
| adrenal 1 | -0,0951 | 0,0248 | 0,0117 | -0,3673 | -0,0190 | -0,1498 | 0,0509 | 0,0457 | 0,2161 | 0,1214 | 0,1902 | 0,5576 | 0,2490 | 0,0285 | -1,9193 | -0,1105 | -0,6513 | 0,1645 |
| adrenal 2 | 0,0093 | -0,1110 | -0,0739 | 0,1225 | 0,0585 | 0,0157 | 0,0447 | 0,1237 | 0,0048 | -0,5705 | 0,0560 | 0,0768 | -0,0951 | -0,0504 | -0,7959 | -3,5471 | -0,2100 | -0,2751 |
| adrenal 3 | -0,0865 | 0,0231 | 0,0017 | -0,2391 | -0,0034 | -0,1385 | 0,0632 | 0,0474 | 0,1524 | 0,1684 | -0,2390 | 0,1597 | 0,1881 | -0,0661 | 0,7682 | -0,2142 | -1,4200 | 0,2074 |
| adrenal 4 | -0,0689 | 0,0486 | -0,0120 | -0,0192 | 0,0055 | -0,0961 | 0,0282 | 0,0600 | 0,2099 | 0,2288 | -0,0779 | 0,0990 | 0,1478 | -0,0178 | -0,0361 | -0,1004 | -0,5519 | 0,1952 |
| bladder 1 | 0,0300 | 0,0263 | -0,0273 | 0,0654 | 0,0597 | 0,0964 | 0,0207 | 0,0160 | -0,0088 | -0,4568 | 0,0330 | -2,1445 | -0,5849 | -0,0747 | -2,5242 | -1,8706 | -0,2580 | -0,3496 |
| bladder 2 | 0,0074 | 0,0279 | 0,0114 | -0,2171 | 0,1020 | 0,1007 | 0,0700 | 0,0140 | -0,0900 | -2,0082 | 0,0212 | -0,7584 | -0,4056 | -0,0492 | -0,8332 | -1,8252 | -0,8596 | -0,1986 |
| bowel 1 | 0,0363 | 0,0153 | -0,0670 | 0,0403 | -0,0844 | 0,0695 | -0,0835 | -0,0337 | 0,1980 | 0,0926 | 0,1459 | -0,5010 | -0,2243 | 0,2043 | -0,6611 | -2,7496 | -0,8099 | -0,3803 |
| bowel 2 | -0,0754 | 0,0125 | 0,0988 | -0,1784 | 0,0391 | 0,0491 | 0,0774 | 0,1889 | 0,0604 | 0,0493 | -0,2823 | -1,4252 | -2,0878 | -0,1370 | -2,0203 | -0,4021 | 0,3053 | -1,0319 |
| bowel 3 | -0,0454 | -0,0545 | 0,1318 | 0,1361 | 0,0473 | 0,0211 | 0,0509 | 0,0740 | 0,0324 | -0,1763 | -0,1186 | -0,0103 | -1,3114 | -0,1167 | -0,2481 | -0,4912 | 0,1006 | -0,3933 |
| brain 1 | 0,0026 | -0,1868 | -0,2073 | 0,0371 | 0,1160 | -0,0014 | -0,1261 | -0,0014 | 0,1071 | 0,2857 | -0,0107 | 0,3462 | 0,1686 | 0,2277 | -0,4773 | -2,9428 | -1,8850 | 0,1136 |
| brain 2 | 0,0692 | -0,1263 | 0,0749 | 0,3403 | 0,0893 | 0,0315 | -0,1704 | -0,0359 | -0,3686 | -1,7678 | 0,0385 | -0,7492 | -0,0768 | -0,2725 | -0,1818 | -1,4634 | -0,7342 | 0,1407 |
| brain 3 | -0,0001 | -0,0190 | 0,0330 | 0,1263 | -0,0857 | -0,0411 | -0,0638 | -0,0380 | -0,0727 | -0,0190 | 0,2196 | -0,4174 | -0,0203 | 0,1596 | -0,3041 | -0,3122 | -0,5936 | 0,2766 |
| brain 4 | 0,0416 | -0,1296 | 0,0255 | 0,0676 | 0,0360 | -0,0190 | -0,0538 | 0,0748 | -0,0872 | -0,4646 | 0,0470 | -1,1955 | 0,1180 | -0,0708 | 0,0748 | -0,5250 | -1,5132 | 0,1445 |
| brain 5 | -0,0145 | -0,0034 | 0,0340 | -0,1579 | 0,0083 | -0,0160 | -0,1591 | -0,1654 | -0,0777 | -0,0034 | 0,1142 | -0,0265 | 0,2287 | 0,3153 | 0,3257 | -0,6870 | -1,6792 | 0,1955 |
| brain 6 | 0,0470 | -0,3377 | -0,1525 | 0,1852 | 0,1038 | 0,0308 | -0,0355 | -0,0906 | 0,0994 | 0,2850 | -0,0266 | -0,0824 | 0,0682 | 0,1616 | -2,5004 | -0,9262 | -0,0364 | -0,1112 |
| brain 7 | 0,0398 | -0,1271 | 0,0328 | 0,1229 | 0,1370 | 0,0563 | -0,1199 | 0,0171 | -0,1839 | -0,1392 | -0,0486 | -0,8730 | 0,0577 | -0,1574 | -0,6506 | -1,8988 | -0,1833 | -0,0213 |
| brain 8 | 0,0674 | -0,1660 | -0,2090 | 0,6583 | 0,1041 | -0,0515 | -0,1368 | -0,0623 | -0,2097 | -2,6845 | -0,0185 | -0,6716 | -0,2409 | 0,0059 | -3,7748 | -4,8113 | -1,0456 | 0,1429 |
| breast | 0,0143 | 0,1224 | 0,0367 | -0,0702 | 0,0364 | -0,0578 | 0,0816 | 0,0089 | 0,0206 | -0,7074 | 0,0399 | -0,7408 | 0,1693 | -0,2251 | -2,3552 | -0,0743 | -1,1502 | 0,0766 |
| cervix 1 | 0,0340 | -0,0026 | 0,0145 | -0,2058 | -0,0615 | 0,1643 | 0,0665 | 0,0234 | -0,3018 | -0,8281 | 0,0999 | -0,9453 | -0,1712 | -0,1179 | -0,7675 | -2,7357 | -0,5677 | -0,2606 |
| cervix 2 | 0,0885 | -0,0508 | -0,0202 | 0,2338 | -0,1673 | 0,1579 | -0,1207 | -0,1621 | -0,1141 | 0,2338 | -0,1801 | -0,6227 | -0,5344 | -0,1037 | -0,8290 | -0,1500 | 0,4928 | -0,2275 |
| cervix 3 | 0,0397 | -0,3968 | 0,1284 | 0,1284 | 0,0263 | 0,1284 | -0,1779 | -0,0959 | -0,3673 | -1,1841 | -0,1960 | -0,1779 | 0,0008 | 0,1126 | -0,9344 | 0,1284 | 0,4225 | -0,5236 |
| colon 1 | 0,1276 | 0,0166 | 0,1190 | -0,4156 | -0,0584 | 0,0362 | 0,0748 | -0,2706 | -0,0979 | 0,2155 | -0,2767 | -0,4675 | -0,6704 | 0,0908 | -0,5321 | -0,0844 | 0,1957 | -0,1672 |
| colon 2 | 0,0445 | -0,3206 | 0,0543 | 0,1573 | -0,1350 | 0,1573 | 0,0055 | -0,0445 | -0,1906 | 0,0132 | 0,0867 | -0,9521 | -1,0546 | -0,2429 | -3,2282 | -0,1433 | 0,4852 | -0,3233 |
| colon 3 | 0,0554 | -0,3801 | 0,0384 | 0,1732 | -0,0949 | 0,1732 | -0,3152 | -0,0398 | -0,0929 | 0,1732 | -0,2628 | -1,6560 | -0,1482 | -0,2415 | 0,2763 | 0,0372 | 0,5902 | -0,5659 |
| diaphr | -0,0659 | 0,0527 | -0,0734 | -0,6833 | 0,0246 | -0,1074 | -0,0010 | 0,0696 | 0,2234 | 0,3637 | -0,3513 | -0,0531 | 0,4445 | 0,0253 | -0,0655 | 0,0515 | -0,0383 | 0,2241 |
| epidyd | -0,0332 | -0,0493 | 0,0148 | -0,2105 | 0,0137 | 0,1453 | 0,0929 | 0,0215 | -0,2357 | -0,8115 | -0,0133 | 0,1810 | 0,0426 | -0,0683 | -3,9104 | -2,2621 | -0,5849 | -0,0362 |
| esoph 1 | -0,0575 | 0,0858 | 0,0178 | -0,0792 | 0,0150 | 0,0585 | -0,0585 | 0,0908 | 0,0461 | -0,3283 | -0,0339 | -0,4052 | 0,0636 | -0,0721 | 0,0717 | -0,1121 | -1,2200 | -0,0425 |
| esoph 2 | 0,0303 | -0,1690 | -0,0864 | 0,1265 | -0,0748 | -0,0083 | 0,0430 | -0,1899 | 0,3041 | 0,3309 | -0,0065 | 0,0781 | -0,0108 | 0,3172 | -0,0880 | -0,8208 | -0,2041 | -0,4898 |
| esoph 3 | -0,0278 | 0,0042 | -0,0757 | 0,0219 | -0,0017 | 0,0900 | 0,0114 | 0,0909 | 0,0201 | -0,9334 | 0,0575 | -1,5612 | 0,1285 | 0,0811 | -0,5917 | -1,7161 | -0,8485 | -0,2436 |
| fallop t 1 | 0,0700 | 0,0531 | 0,0306 | -0,0059 | 0,0444 | 0,0093 | -0,0563 | -0,0362 | -0,0203 | -0,1980 | 0,1402 | -0,5193 | -0,0167 | -0,0447 | -1,9491 | -2,0913 | -0,7947 | -0,1480 |
| fallop t 2 | 0,0613 | 0,0180 | 0,0157 | -0,3252 | 0,0062 | 0,0149 | 0,0125 | -0,0353 | 0,0400 | -0,4757 | 0,1591 | 0,0676 | -0,1130 | 0,0324 | -3,9578 | -1,4237 | -0,7826 | 0,0128 |
| fallop t 3 | 0,0735 | 0,0509 | -0,0920 | 0,0939 | 0,0531 | -0,0006 | 0,1264 | 0,0233 | -0,1234 | -0,3605 | 0,1272 | -1,1255 | -0,7790 | -0,3860 | -0,6792 | -2,7748 | -1,5178 | 0,1015 |
| fallop t 4 | 0,0636 | 0,0733 | -0,0535 | 0,0813 | 0,1129 | 0,0640 | -0,0098 | -0,0986 | -0,0865 | -0,8979 | -0,1984 | -2,1286 | 0,1845 | -0,3262 | -0,5438 | 0,0756 | -0,2077 | -0,1699 |
| gallbl | -0,0869 | 0,0573 | 0,0020 | -0,2049 | 0,0621 | -0,0288 | 0,2776 | 0,0233 | -0,1766 | -0,1766 | 0,1743 | 0,2496 | -0,0338 | -0,5415 | -0,1136 | -1,4623 | -0,1766 | -0,0865 |
| heart 1 | 0,0025 | -1,0708 | -0,3024 | 0,0202 | -0,1431 | -0,1546 | -0,2583 | -0,1561 | 0,2690 | 0,3073 | -0,5184 | 0,0482 | 0,1686 | 0,3295 | 1,2346 | -0,4418 | 0,4569 | -1,2909 |
| heart 2 | 0,0067 | 0,0443 | -0,0228 | 0,2054 | 0,0253 | -0,1275 | -0,0065 | 0,0896 | -0,1173 | 0,0484 | 0,0935 | 0,0045 | -0,1317 | 0,0941 | 0,2415 | -2,2471 | -2,4139 | -0,0023 |
| heart 3 | 0,0420 | 0,1261 | -0,0470 | 0,0300 | 0,0114 | -0,0489 | -0,0395 | 0,0997 | -0,0505 | -0,3419 | -0,0980 | -1,5072 | -0,0499 | -0,3476 | -0,1073 | 0,0049 | -0,1361 | 0,2413 |
| heart 4 | -0,0755 | 0,0544 | -0,0238 | -0,0816 | -0,0388 | -0,1156 | 0,0464 | -0,0596 | 0,3306 | 0,4120 | -0,2560 | 0,0130 | 0,1604 | 0,2403 | 0,5197 | -1,2070 | -0,4168 | -0,0434 |
| heart 5 | 0,0069 | 0,0557 | -0,1326 | 0,4015 | 0,2080 | -0,2595 | -0,0523 | 0,0514 | 0,0183 | -0,1153 | -0,2209 | -0,3383 | 0,2247 | -0,0771 | -0,1474 | -0,9318 | **-**2,8430 | 0,1764 |
| heart 6 | 0,0011 | -0,0003 | -0,3780 | 0,0388 | 0,0476 | -0,4831 | -0,0235 | -0,1742 | 0,3924 | 0,5429 | 0,0388 | 0,4587 | -0,1678 | 0,1027 | 0,6662 | -0,1757 | 0,1829 | 0,3618 |
| kidney 1 | 0,1216 | -0,1236 | 0,0493 | 0,2670 | -0,0502 | 0,1444 | -0,2214 | -0,2320 | -0,3562 | -1,1496 | -0,7228 | -1,2194 | -3,9820 | 0,2670 | 0,2670 | -0,0802 | 0,2670 | -0,8622 |
| kidney 2 | -0,0007 | -0,0334 | 0,0962 | 0,2184 | -0,1103 | -4,1465 | -0,0258 | -0,0415 | 0,2994 | 0,1826 | 0,1464 | 0,6768 | 0,4571 | 0,3575 | -0,5052 | -0,3336 | -0,0875 | -0,1053 |
| kidney 3 | 0,0128 | 0,2271 | 0,1344 | 0,2271 | -0,0292 | -0,2336 | 0,1250 | -0,4254 | 0,1317 | 0,2271 | 0,0851 | 0,2271 | -0,2834 | 0,1028 | -1,1584 | -0,6243 | 0,2271 | -0,4368 |
| kidney 4 | 0,0344 | -0,1366 | 0,0379 | 0,2959 | 0,1169 | 0,0488 | -0,0222 | -0,0717 | -0,2469 | 0,0668 | -0,0997 | -0,1543 | -0,8650 | -0,0522 | 0,1671 | -1,0804 | -0,2464 | -0,1756 |
| kidney 5 | 0,0939 | -0,1232 | 0,0480 | 0,4705 | -0,0227 | -0,1491 | -0,0350 | -0,2980 | 0,0870 | 0,0996 | -0,0906 | 0,1407 | -1,8161 | 0,2172 | -0,4845 | -0,5028 | 0,2428 | -0,0389 |
| liver 1 | -0,0018 | -0,0731 | -0,0025 | -0,3392 | -0,0309 | -0,1283 | 0,0172 | -0,0042 | 0,1716 | 0,3010 | 0,1636 | 0,5769 | 0,2418 | 0,2483 | -0,9280 | -1,2076 | -0,6149 | -0,2298 |
| liver 2 | -0,0171 | -0,0904 | 0,0247 | -0,0385 | 0,0245 | -0,1791 | 0,2497 | -0,7448 | 0,1374 | 0,4214 | 0,0584 | 0,4484 | -0,7743 | 0,1655 | -1,1603 | 0,1957 | 0,5843 | -0,0670 |
| liver 3 | 0,0750 | -0,7190 | 0,0182 | -0,2565 | 0,0871 | -0,1384 | 0,0860 | -0,3037 | -0,1865 | -0,0145 | -1,0311 | 0,1820 | -0,3939 | 0,1956 | -0,8343 | 0,3202 | 1,0475 | -1,1124 |
| liver 4 | -0,0907 | -1,8608 | -0,2203 | -0,1698 | -0,1033 | -0,2867 | -0,0161 | -0,5817 | -0,1319 | 0,0311 | 0,0528 | 0,8196 | 0,3206 | 0,4172 | -1,5804 | 0,8629 | 0,9912 | -0,0978 |
| liver 5 | -0,1345 | -0,0271 | 0,1501 | -0,2357 | 0,0315 | -0,1793 | 0,1771 | -0,1128 | 0,1361 | 0,4926 | 0,0289 | 0,5703 | -0,0437 | 0,2141 | -0,7131 | -1,0414 | -0,2307 | 0,0109 |
| lung 1 | 0,0479 | -0,0886 | -0,0585 | -0,2388 | 0,0772 | -0,0015 | 0,0427 | 0,0206 | 0,1120 | -0,0012 | 0,1342 | -0,0794 | -0,2549 | -0,0092 | -0,1185 | -0,8747 | -0,2573 | -0,2610 |
| lung 2 | 0,0554 | -0,1073 | 0,0066 | -0,3428 | -0,0210 | 0,1248 | -0,2337 | 0,0793 | -0,1061 | -0,3290 | -0,1117 | -1,1702 | -0,5289 | 0,1932 | -1,0340 | 0,0939 | 0,3642 | -0,6000 |
| lung 3 | 0,0785 | -0,0880 | -0,0423 | -0,4248 | -0,0154 | 0,1622 | -0,2302 | 0,0503 | -0,1495 | -0,0211 | -0,1529 | -2,0186 | -0,3310 | 0,0295 | 0,1025 | 0,1010 | 0,2863 | -1,0241 |
| lung 4 | -0,0503 | 0,0539 | 0,0022 | -0,3858 | 0,0312 | -0,0114 | -0,0182 | 0,0549 | -0,0056 | -0,0594 | 0,2748 | 0,2439 | 0,2827 | -0,1403 | -1,3828 | -0,5183 | -1,2627 | 0,0130 |
| muscle 1 | -0,1437 | -0,0505 | -0,0778 | -0,5832 | -0,0369 | -0,1258 | 0,0302 | -0,1239 | 0,4429 | 0,6208 | 0,0311 | 0,2535 | 0,3876 | 0,2775 | 0,1996 | -0,4134 | -1,0290 | 0,0698 |
| muscle 2 | -0,0882 | 0,0519 | -0,0225 | -0,1155 | -0,0466 | -0,1479 | -0,0627 | 0,2140 | 0,1085 | -0,2006 | -0,1264 | -0,3511 | 0,3549 | 0,0673 | -0,0525 | 0,1919 | -0,2422 | 0,2122 |
| ovary 1 | 0,0350 | -0,1722 | 0,1071 | 0,1407 | -0,0082 | 0,0992 | -0,0808 | 0,0388 | -0,3000 | 0,1071 | 0,0266 | -1,0023 | 0,0450 | -0,2040 | -2,0743 | -0,4873 | 0,1071 | -0,4876 |
| ovary 2 | 0,0559 | -0,0247 | -0,0582 | 0,3591 | -0,2498 | 0,1044 | -0,1983 | 0,1023 | 0,0060 | -0,3460 | 0,0289 | 0,0802 | -0,3411 | -0,0546 | -0,7478 | -0,5802 | -0,5818 | -0,0723 |
| ovary 3 | 0,0486 | 0,1490 | 0,0599 | 0,0040 | -0,1450 | 0,0348 | 0,0443 | -0,0745 | -0,0733 | 0,2010 | 0,0963 | -1,4772 | -0,0716 | -0,2279 | -1,3950 | -0,5778 | -2,3060 | 0,1715 |
| ovary 4 | -0,0190 | 0,0875 | 0,0762 | 0,4520 | -0,0515 | 0,1239 | -0,0406 | 0,0771 | -0,3384 | -0,4845 | -0,2805 | -0,3361 | -0,6380 | -1,4311 | 0,0841 | -0,9144 | -2,**9359** | -0,1093 |
| ovary 5 | 0,0662 | 0,0502 | 0,0852 | -0,1057 | -0,0143 | 0,0373 | -0,0133 | 0,0579 | -0,9886 | -0,5323 | 0,1140 | -0,2439 | -0,1107 | -0,6743 | -3,3611 | -0,4701 | 0,0245 | 0,1824 |
| pancre 1 | -0,0063 | 0,0890 | 0,0215 | -0,1721 | 0,0658 | 0,0819 | 0,0359 | 0,0677 | -0,2330 | -0,9035 | -0,0122 | -0,3309 | -0,0419 | -0,2101 | 0,0202 | -1,4333 | -0,9741 | -0,1191 |
| pancre 2 | -0,2478 | 0,0657 | -0,2888 | 0,0657 | -0,7720 | -0,8910 | -0,8568 | -0,7506 | -0,6291 | 0,0657 | -3,1662 | 0,0657 | -1,1204 | -0,5278 | 0,0657 | 1,6185 | 0,8442 | -1,0235 |
| pericard | 0,0580 | -0,1225 | -0,1246 | 0,0582 | -0,1051 | -0,0127 | -0,0255 | -0,0490 | -0,0754 | -0,4473 | 0,2761 | -0,8550 | 0,0371 | -0,3788 | 0,0582 | 0,1222 | 0,7860 | -0,6809 |
| placenta | 0,1085 | 0,0811 | 0,0396 | 0,0444 | 0,0268 | -0,0115 | 0,0271 | -0,0367 | -0,2754 | -1,3722 | 0,0795 | -0,4630 | -0,7560 | -0,0141 | -0,2530 | -0,9210 | -0,7279 | -0,1215 |
| prostate 1 | -0,0339 | 0,1574 | 0,0587 | -0,3542 | -0,0034 | 0,1597 | -0,0580 | 0,0559 | -0,0736 | -1,4902 | 0,1048 | -3,4877 | -0,1643 | -0,6061 | -0,3228 | -0,7227 | -3,2875 | -0,0354 |
| prostate 2 | -0,0002 | 0,1779 | 0,1207 | -0,2691 | -0,0082 | 0,1470 | 0,0228 | -0,0146 | -0,3901 | -2,5594 | 0,0551 | -1,2975 | -0,1829 | -0,5150 | -1,1636 | -0,8869 | -3,2842 | -0,0483 |
| prostate 3 | 0,0688 | 0,1216 | 0,0284 | -0,2393 | 0,0015 | 0,1437 | -0,0432 | -0,0738 | 0,0777 | -1,5940 | 0,0216 | -0,7518 | -1,0587 | -0,3371 | -0,1953 | -3,1403 | -0,5844 | -0,1661 |
| prostate 4 | 0,0025 | 0,0276 | 0,0288 | -0,5862 | -0,0054 | 0,1192 | 0,0410 | 0,0092 | 0,0288 | -0,2774 | 0,0741 | -0,1258 | -0,3313 | -0,0253 | -1,0241 | -1,5663 | -0,5247 | -0,0853 |
| prostate 5 | 0,0506 | 0,0793 | 0,0959 | 0,0978 | 0,0617 | 0,0597 | -0,0154 | -0,1006 | -0,3126 | -0,1214 | 0,0969 | -1,2363 | -0,5175 | -0,2995 | -0,5279 | -2,4839 | -0,6930 | 0,0623 |
| saliv gl 1 | 0,0075 | 0,0749 | 0,0439 | 0,0867 | 0,0073 | -0,0853 | 0,0420 | 0,1397 | -0,2194 | -1,5237 | -0,0822 | -1,6371 | -0,0409 | -0,2644 | -0,2488 | -0,0715 | -0,8044 | 0,3076 |
| saliv gl 2 | 0,0675 | 0,0284 | -0,0150 | -0,2721 | 0,0288 | -0,0212 | 0,0263 | 0,1066 | -0,1921 | 0,0675 | -0,0229 | -2,2836 | -0,2079 | -0,2768 | -0,3077 | -0,0259 | -0,5718 | 0,2019 |
| saliv gl 3 | -0,0057 | 0,1186 | 0,1181 | -0,1819 | -0,0163 | -0,0172 | -0,1163 | 0,1740 | -0,5697 | -1,1548 | -0,0562 | -0,6286 | -0,2532 | -0,0846 | -0,2736 | -0,0500 | -0,7796 | 0,3015 |
| saliv gl 4 | -0,1590 | 0,2059 | 0,0976 | 0,0278 | 0,0446 | -0,0151 | 0,0113 | 0,1084 | -0,3609 | -0,9858 | -0,8639 | -0,1391 | -0,0131 | -0,3935 | 0,4882 | 0,1097 | -0,0563 | 0,3093 |
| sem ves 1 | 0,0396 | 0,0408 | -0,0403 | -0,0880 | 0,0353 | 0,0161 | -0,0344 | 0,1683 | -0,4329 | -2,3501 | -0,0197 | -0,2740 | -0,0314 | -0,1294 | 0,2881 | -1,3078 | -3,6324 | 0,0225 |
| sem ves 2 | -0,0492 | -0,0847 | -0,0092 | -0,0092 | 0,1259 | 0,0780 | -0,0858 | 0,1254 | -0,0783 | -0,3354 | -0,0092 | -0,0092 | -0,0092 | -0,1387 | -0,0092 | -0,0092 | -1,1704 | -0,3223 |
| sem ves 3 | -0,0319 | 0,0137 | -0,1255 | -0,0970 | 0,0769 | -0,0177 | 0,0194 | 0,1176 | 0,0751 | 0,0324 | -0,0080 | 0,1799 | -0,0166 | 0,0494 | -0,4610 | -0,1255 | -2,1353 | 0,0136 |
| stomach 1 | 0,0995 | -0,0415 | -0,0649 | -0,0378 | -0,0682 | 0,0373 | -0,0376 | -0,0104 | 0,0325 | -0,2068 | -0,0629 | 0,1214 | 0,0421 | 0,1623 | -2,7659 | -0,9238 | -0,5780 | -0,0343 |
| stomach 2 | 0,0711 | -0,0302 | 0,0310 | 0,0629 | -0,0263 | 0,0934 | -0,0393 | -0,0626 | -0,4539 | -1,2011 | -0,0445 | -0,5839 | -0,2844 | -0,1549 | 0,0303 | 0,3116 | 0,4163 | -0,7925 |
| stomach 3 | 0,0903 | 0,0405 | 0,1781 | -0,3054 | 0,0312 | 0,0863 | -0,1140 | 0,0650 | -0,7088 | -1,8708 | -0,0872 | -1,9793 | -0,5698 | -0,4187 | -1,7570 | -0,3481 | 0,2581 | -0,3531 |
| stomach 4 | 0,0156 | 0,0578 | 0,1099 | 0,0295 | -0,0476 | 0,0578 | 0,0637 | -0,0200 | 0,0578 | -0,5535 | -0,0948 | 0,0578 | -0,8848 | -0,0304 | -1,9298 | -1,3816 | -0,0679 | -0,1713 |
| testis 1 | -0,0911 | 0,1378 | 0,0777 | -0,3061 | -0,0440 | 0,0362 | 0,1097 | 0,0923 | -0,2337 | -1,0567 | 0,0989 | -0,0662 | -0,1085 | -0,2212 | -2,3143 | -0,0206 | -0,5003 | 0,2129 |
| testis 2 | -0,1305 | 0,0210 | 0,0081 | -0,0303 | -0,0300 | 0,1345 | 0,0065 | -0,1215 | -0,7953 | 0,1875 | 0,0039 | -4,3315 | 0,0592 | -0,3400 | 0,5557 | 0,1911 | -0,0460 | 0,3629 |
| testis 3 | -0,1006 | -0,0189 | -0,0625 | -0,0261 | -0,1755 | 0,0442 | -0,0502 | 0,3035 | -0,8225 | -0,0261 | -0,1734 | -0,0261 | -1,1625 | -0,0281 | 0,4472 | -0,1285 | -0,4572 | 0,3848 |
| uterus 1 | 0,0167 | 0,0046 | 0,0123 | -0,0130 | -0,0480 | 0,0845 | -0,0554 | -0,0154 | 0,0747 | -0,4973 | 0,1182 | -0,2942 | 0,0436 | 0,1001 | -0,9461 | -1,3686 | -2,0030 | -0,1290 |
| uterus 2 | 0,0827 | 0,1132 | -0,0440 | 0,0765 | -0,0625 | 0,1074 | 0,0248 | 0,0765 | -0,1515 | -0,7702 | -0,1787 | -0,9970 | -0,4578 | -0,3310 | -0,1269 | -1,6607 | -0,7359 | -0,1169 |
| uterus 3 | 0,0819 | 0,0133 | -0,1381 | 0,1801 | -0,0119 | 0,1801 | -0,0627 | -0,1490 | -0,7452 | 0,1801 | -0,1377 | -0,2739 | 0,1801 | -0,5497 | 0,1801 | -0,0447 | 0,1344 | -0,6271 |
| uterus 4 | 0,1153 | 0,0907 | 0,0308 | -0,0049 | -0,0864 | 0,1620 | -0,0383 | -0,3907 | -0,4322 | 0,1620 | 0,1296 | -1,1082 | -2,8828 | -0,0683 | -0,2132 | -0,7559 | -0,8074 | -0,0039 |
| uterus 5 | 0,0365 | -0,0169 | -0,0537 | 0,2115 | -0,0399 | 0,0583 | 0,0679 | -0,1184 | -0,0012 | -1,9158 | 0,0843 | -0,4845 | -0,3321 | 0,0370 | 0,1839 | -0,0012 | -0,7735 | -0,1604 |
| vagina | 0,1184 | -0,2780 | 0,1119 | 0,0880 | -0,2861 | 0,1439 | -0,2448 | -0,2021 | -0,3620 | 0,3823 | -0,2526 | -1,5405 | 0,0950 | -0,1684 | 0,2612 | 0,1796 | 0,5437 | -0,3009 |
