# Supplementary material for: Epigenetic hereditary transcription profiles II, aging revisited
Source: Biol Direct. 2007 Dec 28;2:39. doi: 10.1186/1745-6150-2-39 (PMC2265679; doi:10.1186/1745-6150-2-39)
Supplement: Additional file 2 — Values for "log obs/exp" after removal of the variation due to proteasome expression level (= "2nd log obs/exp"). Second treatment of the data. [file 1745-6150-2-39-S2.doc]

Additional file 2. Values for “log obs/exp” after removal of the variation due to proteasome expression level (= “2^nd^ log obs/exp”)

|  | 2nd log obs/exp | |  |  |  |  |  |  |  |  |  |  |  |  |  |  |  |  |
| --- | --- | --- | --- | --- | --- | --- | --- | --- | --- | --- | --- | --- | --- | --- | --- | --- | --- | --- |
| tissue | A1 | A2a | A2b | A3a | A3b | A4 | A5 | A6 | A7a | A7b | B2 | B3 | B5 | B6 | B7a | B7b | B7c | B7d |
| adrenal 1 | 0,0154 | -0,0544 | 0,0488 | 0,0247 | -0,0359 | -0,0508 | -0,0307 | -0,0301 | 0,0530 | 0,0822 | 0,1981 | 0,4824 | -0,0903 | 0,0250 | -1,6831 | 0,3351 | 0,6662 | -0,0826 |
| adrenal 2 | -0,0283 | -0,0520 | -0,0762 | 0,1098 | 0,0613 | -0,0235 | 0,0804 | 0,1467 | 0,1577 | -0,0766 | 0,1034 | 0,7717 | 0,2172 | 0,0448 | 0,0227 | -2,6178 | 0,2288 | -0,0779 |
| adrenal 3 | -0,0503 | 0,0133 | 0,0190 | -0,0504 | -0,0104 | -0,1089 | 0,0525 | 0,0212 | 0,1480 | 0,3970 | -0,2112 | 0,4715 | 0,1762 | -0,0200 | 1,2970 | 0,4744 | -0,5440 | 0,1834 |
| adrenal 4 | -0,0291 | 0,0354 | 0,0063 | 0,1795 | -0,0020 | -0,0631 | 0,0032 | 0,0314 | 0,1977 | 0,4442 | -0,0511 | 0,3916 | 0,1197 | 0,0259 | 0,4783 | 0,5762 | 0,3458 | 0,1603 |
| bladder 1 | -0,0040 | 0,0820 | -0,0287 | 0,0626 | 0,0620 | 0,0605 | 0,0513 | 0,0365 | 0,1363 | 0,0241 | 0,0794 | -1,4684 | -0,2885 | 0,0181 | -1,7198 | -0,9531 | 0,2023 | -0,1634 |
| bladder 2 | -0,0059 | 0,0642 | 0,0155 | -0,1635 | 0,1015 | 0,0841 | 0,0874 | 0,0208 | 0,0111 | -1,6016 | 0,0621 | -0,1897 | -0,2001 | 0,0299 | -0,1100 | -0,9752 | -0,2768 | -0,0743 |
| bowel 1 | 0,0270 | 0,0479 | -0,0618 | 0,1050 | -0,0854 | 0,0566 | -0,0705 | -0,0296 | 0,2905 | 0,4846 | 0,1857 | 0,0468 | -0,0366 | 0,2807 | 0,0462 | -1,9128 | -0,2032 | -0,2681 |
| bowel 2 | -0,1325 | 0,0897 | 0,0912 | -0,2445 | 0,0445 | -0,0083 | 0,1298 | 0,2249 | 0,2549 | 0,6135 | -0,2297 | -0,6288 | -1,6897 | -0,0287 | -1,1248 | 0,5910 | 0,6283 | -0,7762 |
| bowel 3 | -0,0643 | -0,0129 | 0,1344 | 0,1744 | 0,0476 | -0,0007 | 0,0728 | 0,0845 | 0,1454 | 0,2504 | -0,0762 | 0,5875 | -1,0813 | -0,0339 | 0,4971 | 0,3771 | 0,6502 | -0,2522 |
| brain 1 | -0,0119 | -0,1493 | -0,2035 | 0,0874 | 0,1157 | -0,0191 | -0,0884 | 0,0062 | 0,2107 | 0,6965 | 0,0305 | 0,9211 | 0,3794 | 0,3076 | 0,2506 | -2,0888 | -1,3093 | 0,2415 |
| brain 2 | -0,0026 | -0,0353 | 0,0634 | 0,2340 | 0,0966 | -0,0397 | -0,1062 | 0,0098 | -0,1428 | -1,1507 | 0,0950 | 0,1237 | 0,3860 | -0,1544 | 0,7715 | -0,4224 | -0,4984 | 0,4405 |
| brain 3 | -0,0090 | 0,0132 | 0,0383 | 0,1921 | -0,0868 | -0,0535 | -0,0525 | -0,0342 | 0,0189 | 0,3715 | 0,2593 | 0,1281 | 0,1655 | 0,2357 | 0,4015 | 0,5232 | 0,0156 | 0,3875 |
| brain 4 | 0,0051 | -0,0716 | 0,0234 | 0,0580 | 0,0387 | -0,0572 | -0,0163 | 0,0970 | 0,0632 | 0,0253 | 0,0941 | -0,5065 | 0,4253 | 0,0237 | 0,8890 | 0,4006 | -1,0677 | 0,3382 |
| brain 5 | -0,0284 | 0,0335 | 0,0380 | -0,1058 | 0,0079 | -0,0331 | -0,1431 | -0,1583 | 0,0246 | 0,4052 | 0,1552 | 0,5452 | 0,4367 | 0,3948 | 1,0511 | 0,1648 | -1,0998 | 0,3215 |
| brain 6 | 0,0068 | -0,2763 | -0,1556 | 0,1653 | 0,1070 | -0,0108 | 0,0023 | -0,0659 | 0,2579 | 0,7884 | 0,0215 | 0,6261 | 0,3920 | 0,2586 | -1,6714 | 0,0116 | 0,3868 | 0,0938 |
| brain 7 | -0,0255 | -0,0423 | 0,0231 | 0,0345 | 0,1435 | -0,0087 | -0,0609 | 0,0584 | 0,0279 | 0,4542 | 0,0062 | -0,0343 | 0,4917 | -0,0437 | 0,2768 | -0,8791 | 0,0914 | 0,2588 |
| brain 8 | -0,0002 | -0,0790 | -0,2194 | 0,5635 | 0,1109 | -0,1188 | -0,0795 | -0,0194 | 0,0072 | -2,0824 | 0,0369 | 0,1795 | 0,2035 | 0,1212 | -2,8380 | -3,7839 | -0,7850 | 0,4301 |
| breast | 0,0187 | 0,1423 | 0,0455 | 0,0318 | 0,0336 | -0,0578 | 0,0952 | 0,0039 | 0,0839 | -0,3646 | 0,0761 | -0,2642 | 0,2969 | -0,1579 | -1,7017 | 0,7178 | -0,4623 | 0,1477 |
| cervix 1 | 0,0014 | 0,0518 | 0,0135 | -0,2050 | -0,0594 | 0,1297 | 0,0982 | 0,0431 | -0,1595 | -0,3520 | 0,1460 | -0,2760 | 0,1193 | -0,0259 | 0,0317 | -1,8226 | -0,0996 | -0,0783 |
| cervix 2 | 0,0169 | 0,0400 | -0,0316 | 0,1282 | -0,1600 | 0,0870 | -0,0584 | -0,1165 | 0,1112 | 0,8500 | -0,1237 | 0,2488 | -0,0727 | 0,0142 | 0,1233 | 0,8902 | 0,7300 | 0,0715 |
| cervix 3 | -0,0100 | -0,3264 | 0,1228 | 0,0824 | 0,0307 | 0,0778 | -0,1121 | -0,0649 | -0,1885 | -0,6464 | -0,1454 | 0,5802 | 0,3666 | 0,2160 | -0,0680 | 1,0974 | 0,7891 | -0,2900 |
| colon 1 | 0,0775 | 0,0873 | 0,1133 | -0,4626 | -0,0539 | -0,0147 | 0,1217 | -0,2392 | 0,0817 | 0,7545 | -0,2259 | 0,2926 | -0,3029 | 0,1944 | 0,3359 | 0,8858 | 0,5600 | 0,0676 |
| colon 2 | -0,0118 | -0,2442 | 0,0469 | 0,0935 | -0,1297 | 0,1007 | 0,0679 | -0,0091 | 0,0021 | 0,5744 | 0,1390 | -0,1601 | -0,6601 | -0,1352 | -2,3361 | 0,8470 | 0,8131 | -0,0701 |
| colon 3 | -0,0042 | -0,3005 | 0,0302 | 0,1001 | -0,0892 | 0,1134 | -0,2501 | -0,0021 | 0,1071 | 0,7467 | -0,2095 | -0,8463 | 0,2613 | -0,1316 | 1,1818 | 1,0386 | 0,8980 | -0,3025 |
| diaphr | 0,0886 | -0,0675 | -0,0246 | -0,1713 | 0,0018 | 0,0325 | -0,1384 | -0,0354 | -0,0334 | 0,1664 | -0,3552 | -0,3567 | -0,0880 | -0,0074 | -0,0021 | 0,3536 | 1,5397 | -0,1548 |
| epidyd | -0,0703 | 0,0092 | 0,0125 | -0,2219 | 0,0164 | 0,1066 | 0,1281 | 0,0441 | -0,0839 | -0,3193 | 0,0340 | 0,8733 | 0,3527 | 0,0266 | -3,0937 | -1,3344 | -0,1431 | 0,1594 |
| esoph 1 | -0,0260 | 0,0804 | 0,0339 | 0,0968 | 0,0086 | 0,0837 | -0,0769 | 0,0677 | 0,0516 | -0,0830 | -0,0050 | -0,0694 | 0,0720 | -0,0229 | 0,6187 | 0,5916 | -0,3715 | -0,0526 |
| esoph 2 | 0,0061 | -0,1225 | -0,0852 | 0,1503 | -0,0738 | -0,0350 | 0,0692 | -0,1759 | 0,4284 | 0,7766 | 0,0373 | 0,7034 | 0,2425 | 0,4035 | 0,6779 | 0,0647 | 0,3142 | -0,3329 |
| esoph 3 | -0,0310 | 0,0311 | -0,0689 | 0,1033 | -0,0035 | 0,0829 | 0,0207 | 0,0909 | 0,0995 | -0,5634 | 0,0957 | -1,0453 | 0,2893 | 0,1534 | 0,0915 | -0,8992 | -0,2055 | -0,1498 |
| fallop t 1 | 0,0435 | 0,1018 | 0,0311 | 0,0117 | 0,0457 | -0,0196 | -0,0283 | -0,0206 | 0,1089 | 0,2560 | 0,1846 | 0,1179 | 0,2467 | 0,0431 | -1,1741 | -1,1983 | -0,2900 | 0,0158 |
| fallop t 2 | 0,0373 | 0,0643 | 0,0169 | -0,3007 | 0,0072 | -0,0116 | 0,0372 | -0,0214 | 0,1638 | -0,0307 | 0,2029 | 0,6917 | 0,1394 | 0,1186 | -3,1927 | -0,5388 | -0,2630 | 0,1691 |
| fallop t 3 | 0,0520 | 0,0949 | -0,0901 | 0,1251 | 0,0538 | -0,0248 | 0,1492 | 0,0355 | -0,0049 | 0,0756 | 0,1703 | -0,5142 | -0,5374 | -0,3014 | 0,0762 | -1,8980 | -0,9836 | 0,2504 |
| fallop t 4 | 0,0255 | 0,1329 | -0,0560 | 0,0670 | 0,1158 | 0,0242 | 0,0245 | -0,0753 | 0,0676 | -0,4019 | -0,1509 | -1,4307 | 0,4993 | -0,2305 | 0,2771 | 1,0068 | 0,2276 | 0,0289 |
| gallbl | -0,0336 | 0,0315 | 0,0238 | 0,0307 | 0,0527 | 0,0168 | 0,2347 | -0,0143 | -0,2176 | -0,0098 | 0,1974 | 0,4720 | -0,1214 | -0,5068 | 0,3476 | -0,8298 | 0,8013 | -0,1620 |
| heart 1 | -0,0186 | -1,0271 | -0,3004 | 0,0525 | -0,1425 | -0,1785 | -0,2377 | -0,1441 | 0,3867 | 0,7420 | -0,4754 | 0,6576 | 0,4085 | 0,4137 | 1,9885 | 0,4337 | 0,9933 | -1,1432 |
| heart 2 | 0,0340 | 0,0428 | -0,0079 | 0,3698 | 0,0194 | -0,1063 | -0,0240 | 0,0694 | -0,1026 | 0,3091 | 0,1236 | 0,3625 | -0,1045 | 0,1462 | 0,8053 | -1,5294 | -1,5906 | 0,0004 |
| heart 3 | 0,0596 | 0,1336 | -0,0346 | 0,1681 | 0,0068 | -0,0367 | -0,0467 | 0,0859 | -0,0154 | -0,0466 | -0,0653 | -1,0993 | 0,0196 | -0,2891 | 0,4944 | 0,7539 | 0,6301 | 0,2729 |
| heart 4 | -0,0623 | 0,0661 | -0,0126 | 0,0444 | -0,0428 | -0,1075 | 0,0384 | -0,0704 | 0,3751 | 0,7233 | -0,2222 | 0,4440 | 0,2494 | 0,3017 | 1,1387 | -0,4435 | 0,3231 | 0,0014 |
| heart 5 | 0,0333 | 0,0550 | -0,1179 | 0,5636 | 0,2022 | -0,2391 | -0,0679 | 0,0317 | 0,0346 | 0,1484 | -0,1905 | 0,0240 | 0,2555 | -0,0245 | 0,4196 | -0,2115 | -2,0247 | 0,1816 |
| heart 6 | -0,0255 | 0,0484 | -0,3774 | 0,0562 | 0,0489 | -0,5120 | 0,0053 | -0,1586 | 0,5218 | 0,9972 | 0,0832 | 1,0963 | 0,0960 | 0,1906 | 1,4415 | 0,7176 | 0,6871 | 0,5259 |
| kidney 1 | 0,0443 | -0,0274 | 0,0363 | 0,1455 | -0,0421 | 0,0680 | -0,1500 | -0,1825 | -0,1184 | -0,5123 | -0,6648 | -0,3175 | -3,4946 | 0,3888 | 1,2423 | 0,9791 | 0,4696 | -0,5457 |
| kidney 2 | -0,0162 | 0,0050 | 0,0997 | 0,2661 | -0,1104 | -4,1651 | -0,0078 | -0,0333 | 0,4051 | 0,5969 | 0,1879 | 1,2568 | 0,6721 | 0,4380 | 0,2266 | 0,5235 | 0,4824 | 0,0255 |
| kidney 3 | -0,0575 | 0,3167 | 0,1233 | 0,1249 | -0,0220 | -0,3034 | 0,1817 | -0,3807 | 0,3544 | 0,8389 | 0,1413 | 1,0922 | 0,1729 | 0,2199 | -0,2110 | 0,4119 | 0,4717 | -0,1415 |
| kidney 4 | -0,0174 | -0,0643 | 0,0318 | 0,2442 | 0,1216 | -0,0037 | 0,0261 | -0,0393 | -0,0637 | 0,6121 | -0,0485 | 0,6148 | -0,4899 | 0,0526 | 1,0419 | -0,1045 | 0,1078 | 0,0643 |
| kidney 5 | 0,0414 | -0,0501 | 0,0416 | 0,4168 | -0,0179 | -0,2023 | 0,0124 | -0,2650 | 0,2718 | 0,6475 | -0,0393 | 0,9135 | -1,4379 | 0,3224 | 0,3931 | 0,4754 | 0,5927 | 0,2032 |
| liver 1 | 0,0466 | -0,0942 | 0,0180 | -0,1171 | -0,0395 | -0,0874 | -0,0147 | -0,0385 | 0,1411 | 0,4857 | 0,1881 | 0,8251 | 0,1761 | 0,2863 | -0,4473 | -0,5590 | 0,3337 | -0,2905 |
| liver 2 | -0,0093 | -0,0737 | 0,0344 | 0,0727 | 0,0212 | -0,1760 | 0,2467 | -0,7521 | 0,1936 | 0,7521 | 0,0937 | 0,9076 | -0,6615 | 0,2306 | -0,5200 | 0,9770 | 1,2921 | -0,0059 |
| liver 3 | 0,0548 | -0,6763 | 0,0205 | -0,2216 | 0,0876 | -0,1613 | 0,1089 | -0,2924 | -0,0709 | 0,4167 | -0,9884 | 0,7862 | -0,1584 | 0,2792 | -0,0843 | 1,1925 | 1,5898 | -0,9676 |
| liver 4 | -0,0692 | -1,8570 | -0,2069 | -0,0209 | -0,1084 | -0,2708 | -0,0219 | -0,5982 | -0,1052 | 0,3121 | 0,0844 | 1,2070 | 0,3726 | 0,4730 | -0,9944 | 1,5990 | 1,7810 | -0,0781 |
| liver 5 | -0,1048 | -0,0309 | 0,1656 | -0,0645 | 0,0253 | -0,1557 | 0,1601 | -0,1348 | 0,1453 | 0,7442 | 0,0584 | 0,9151 | -0,0276 | 0,2644 | -0,1592 | -0,3320 | 0,6075 | 0,0061 |
| lung 1 | 0,0266 | -0,0449 | -0,0566 | -0,2069 | 0,0778 | -0,0255 | 0,0712 | 0,0326 | 0,2300 | 0,4338 | 0,1772 | 0,5305 | -0,0146 | 0,0751 | 0,6359 | 0,0012 | 0,2785 | -0,1130 |
| lung 2 | -0,0083 | -0,0238 | -0,0028 | -0,4271 | -0,0147 | 0,0611 | -0,1753 | 0,1197 | 0,1026 | 0,2592 | -0,0574 | -0,3391 | -0,1014 | 0,3059 | -0,1123 | 1,1087 | 0,6476 | -0,3243 |
| lung 3 | 0,0151 | -0,0049 | -0,0516 | -0,5082 | -0,0091 | 0,0988 | -0,1739 | 0,0904 | 0,0584 | 0,5658 | -0,0986 | -1,1894 | 0,0949 | 0,1420 | 1,0227 | 1,1146 | 0,5720 | -0,7495 |
| lung 4 | 0,0271 | 0,0056 | 0,0305 | -0,0842 | 0,0186 | 0,0567 | -0,0733 | 0,0012 | -0,0981 | 0,0206 | 0,2915 | 0,3409 | 0,0891 | -0,1217 | -1,0163 | 0,0354 | -0,1417 | -0,1348 |
| muscle 1 | 0,0013 | -0,1619 | -0,0315 | -0,0970 | -0,0585 | 0,0053 | -0,0790 | -0,2227 | 0,2063 | 0,4575 | 0,0298 | -0,0009 | -0,1034 | 0,2510 | 0,3002 | -0,0804 | 0,4929 | -0,2808 |
| muscle 2 | -0,0156 | 0,0081 | 0,0045 | 0,1731 | -0,0585 | -0,0842 | -0,1141 | 0,1634 | 0,0261 | -0,1035 | -0,1084 | -0,2294 | 0,1821 | 0,0891 | 0,3325 | 0,7611 | 0,8507 | 0,0786 |
| ovary 1 | -0,0097 | -0,1066 | 0,1029 | 0,1086 | -0,0045 | 0,0534 | -0,0333 | 0,0665 | -0,1320 | 0,6266 | 0,0759 | -0,2706 | 0,3884 | -0,1040 | -1,2278 | 0,4651 | 0,5039 | -0,2692 |
| ovary 2 | 0,0180 | 0,0346 | -0,0606 | 0,3455 | -0,2469 | 0,0649 | -0,1610 | 0,1254 | 0,1594 | 0,1490 | 0,0764 | 0,7767 | -0,0276 | 0,0408 | 0,0720 | 0,3500 | -0,1448 | 0,1257 |
| ovary 3 | 0,0367 | 0,1841 | 0,0643 | 0,0613 | -0,1456 | 0,0195 | 0,0606 | -0,0687 | 0,0249 | 0,6027 | 0,1368 | -0,9155 | 0,1280 | -0,1498 | -0,6771 | 0,2678 | -1,7152 | 0,2918 |
| ovary 4 | -0,0579 | 0,1477 | 0,0734 | 0,4357 | -0,0485 | 0,0835 | -0,0078 | 0,1009 | -0,1828 | 0,0141 | -0,2327 | 0,3656 | -0,3200 | -1,3350 | 0,9079 | 0,0192 | -2,5048 | 0,0917 |
| ovary 5 | 0,0439 | 0,0950 | 0,0868 | -0,0769 | -0,0135 | 0,0123 | 0,0071 | 0,0707 | -0,8681 | -0,0931 | 0,1574 | 0,3720 | 0,1347 | -0,5892 | -2,6022 | 0,4096 | 0,5534 | 0,3340 |
| pancre 1 | -0,0274 | 0,1326 | 0,0236 | -0,1399 | 0,0664 | 0,0580 | 0,0555 | 0,0796 | -0,1153 | -0,4688 | 0,0308 | 0,2785 | 0,1979 | -0,1258 | 0,7742 | -0,5577 | -0,4377 | 0,0287 |
| pancre 2 | -0,2818 | 0,1214 | -0,2902 | 0,0627 | -0,7697 | -0,9270 | -0,8558 | -0,7301 | -0,4839 | 0,5468 | -3,1198 | 0,7421 | -0,8237 | -0,4349 | 0,8703 | 2,5362 | 1,3041 | -0,8370 |
| pericard | 0,0260 | -0,0687 | -0,1255 | 0,0607 | -0,1031 | -0,0467 | 0,0021 | -0,0297 | 0,0656 | 0,0266 | 0,3220 | -0,1890 | 0,3249 | -0,2873 | 0,8550 | 1,0333 | 1,2579 | -0,5005 |
| placenta | 0,0804 | 0,1313 | 0,0397 | 0,0574 | 0,0283 | -0,0420 | 0,0543 | -0,0200 | -0,1426 | -0,9121 | 0,1244 | 0,1829 | -0,4852 | 0,0749 | 0,5286 | -0,0224 | -0,2332 | 0,0473 |
| prostate 1 | -0,0237 | 0,1718 | 0,0690 | -0,2364 | -0,0070 | 0,1651 | -0,0693 | 0,0470 | -0,0227 | -1,1682 | 0,1394 | -3,0411 | -0,0621 | -0,5427 | 0,3080 | 0,0506 | -2,5653 | 0,0184 |
| prostate 2 | 0,0087 | 0,1935 | 0,1308 | -0,1547 | -0,0116 | 0,1512 | 0,0200 | -0,0227 | -0,3366 | -2,2330 | 0,0901 | -0,8446 | -0,0754 | -0,4507 | -0,5280 | -0,1096 | -2,5693 | 0,0092 |
| prostate 3 | 0,0315 | 0,1803 | 0,0261 | -0,2510 | 0,0042 | 0,1048 | -0,0140 | -0,0511 | 0,2298 | -1,1014 | 0,0688 | -0,0588 | -0,7480 | -0,2421 | 0,6220 | -2,2122 | -0,1434 | 0,0299 |
| prostate 4 | -0,0153 | 0,0682 | 0,0317 | -0,5449 | -0,0052 | 0,0984 | 0,0652 | 0,0190 | 0,1395 | 0,1454 | 0,1162 | 0,4663 | -0,1060 | 0,0568 | -0,2832 | -0,7016 | 0,0314 | 0,0525 |
| prostate 5 | 0,0129 | 0,1384 | 0,0935 | 0,0849 | 0,0645 | 0,0204 | 0,0220 | -0,0776 | -0,1596 | 0,3728 | 0,1443 | -0,5411 | -0,2050 | -0,2042 | 0,2910 | -1,5544 | -0,2546 | 0,2596 |
| saliv gl 1 | -0,0320 | 0,1358 | 0,0410 | 0,0686 | 0,0104 | -0,1264 | 0,0787 | 0,1639 | -0,0623 | -1,0227 | -0,0343 | -0,9320 | 0,2800 | -0,1678 | 0,5777 | 0,8642 | -0,3773 | 0,5106 |
| saliv gl 2 | 0,0330 | 0,0846 | -0,0165 | -0,2765 | 0,0312 | -0,0575 | 0,0756 | 0,1275 | -0,0458 | 0,5504 | 0,0236 | -1,6046 | 0,0909 | -0,1836 | 0,4989 | 0,8934 | -0,1149 | 0,3898 |
| saliv gl 3 | -0,0621 | 0,1951 | 0,1107 | -0,2459 | -0,0110 | -0,0739 | -0,0645 | 0,2095 | -0,3768 | -0,5934 | -0,0038 | 0,1638 | 0,1416 | 0,0232 | 0,6188 | 0,9406 | -0,4521 | 0,5549 |
| saliv gl 4 | -0,1226 | 0,1959 | 0,1149 | 0,2173 | 0,0375 | 0,0148 | -0,0133 | 0,0820 | -0,3659 | -0,7583 | -0,8363 | 0,1710 | -0,0264 | -0,3476 | 1,0159 | 0,7973 | 0,8215 | 0,2844 |
| sem ves 1 | 0,0280 | 0,0756 | -0,0358 | -0,0300 | 0,0346 | 0,0011 | -0,0207 | 0,1740 | -0,3353 | -1,9495 | 0,0208 | 0,2862 | 0,1668 | -0,0514 | 1,0048 | -0,4632 | -3,0399 | 0,1419 |
| sem ves 2 | -0,0612 | -0,0495 | -0,0048 | 0,0479 | 0,1253 | 0,0626 | -0,1093 | 0,1313 | 0,0201 | 0,0666 | 0,0314 | 0,5529 | 0,1907 | -0,0605 | 0,7090 | 0,8367 | -0,5800 | -0,2018 |
| sem ves 3 | -0,0013 | 0,0091 | -0,1097 | 0,0767 | 0,0706 | 0,0068 | 0,0018 | 0,0950 | 0,0825 | 0,2808 | 0,0212 | 0,5201 | -0,0044 | 0,0992 | 0,0894 | 0,5810 | -1,2918 | 0,0061 |
| stomach 1 | 0,0792 | 0,0014 | -0,0627 | -0,0034 | -0,0677 | 0,0142 | -0,0157 | 0,0011 | 0,1486 | 0,2251 | -0,0201 | 0,7267 | 0,2785 | 0,2460 | -2,0150 | -0,0508 | -0,0370 | 0,1111 |
| stomach 2 | 0,0122 | 0,0486 | 0,0229 | -0,0080 | -0,0207 | 0,0343 | 0,0146 | -0,0255 | -0,2557 | -0,6306 | 0,0085 | 0,2216 | 0,1214 | -0,0455 | 0,9326 | 1,3104 | 0,7289 | -0,5316 |
| stomach 3 | 0,0194 | 0,1307 | 0,1668 | -0,4093 | 0,0385 | 0,0159 | -0,0517 | 0,1102 | -0,4847 | -1,2567 | -0,0310 | -1,1109 | -0,1107 | -0,3012 | -0,8071 | 0,6902 | 0,4988 | -0,0559 |
| stomach 4 | -0,0163 | 0,1115 | 0,1090 | 0,0323 | -0,0455 | 0,0239 | 0,1073 | -0,0009 | 0,1985 | -0,0800 | -0,0490 | 0,7231 | -0,5975 | 0,0610 | -1,1335 | -0,4708 | 0,4047 | 0,0088 |
| testis 1 | -0,0574 | 0,1303 | 0,0943 | -0,1239 | -0,0508 | 0,0635 | 0,0894 | 0,0677 | -0,2330 | -0,8195 | 0,1272 | 0,2578 | -0,1100 | -0,1734 | -1,7762 | 0,6757 | 0,3617 | 0,1961 |
| testis 2 | -0,1015 | 0,0179 | 0,0235 | 0,1389 | -0,0361 | 0,1574 | -0,0080 | -0,1429 | -0,7844 | 0,4418 | 0,0335 | -3,9826 | 0,0787 | -0,2891 | 1,1127 | 0,9030 | 0,7876 | 0,3604 |
| testis 3 | -0,1071 | 0,0111 | -0,0566 | 0,0461 | -0,1768 | 0,0339 | -0,0436 | 0,3057 | -0,7360 | 0,3560 | -0,1343 | 0,5073 | -0,9869 | 0,0465 | 1,1437 | 0,6994 | 0,1659 | 0,4888 |
| uterus 1 | 0,0259 | 0,0199 | 0,0224 | 0,1022 | -0,0514 | 0,0890 | -0,0560 | -0,0236 | 0,1276 | -0,1720 | 0,1531 | 0,1573 | 0,1498 | 0,1642 | -0,3116 | -0,5923 | -1,2864 | -0,0723 |
| uterus 2 | 0,0458 | 0,1716 | -0,0462 | 0,0656 | -0,0598 | 0,0688 | 0,0678 | 0,0990 | -0,0001 | -0,2787 | -0,1315 | -0,3056 | -0,1486 | -0,2362 | 0,6891 | -0,7337 | -0,2931 | 0,0781 |
| uterus 3 | 0,0208 | 0,0943 | -0,1467 | 0,1031 | -0,0060 | 0,1190 | 0,0010 | -0,1104 | -0,5422 | 0,7588 | -0,0841 | 0,5433 | 0,5959 | -0,4388 | 1,0913 | 0,9615 | 0,4336 | -0,3594 |
| uterus 4 | 0,0580 | 0,1681 | 0,0232 | -0,0715 | -0,0810 | 0,1044 | 0,0263 | -0,3546 | -0,2373 | 0,7269 | 0,1823 | -0,3109 | -2,4839 | 0,0401 | 0,6829 | 0,2377 | -0,4855 | 0,2523 |
| uterus 5 | 0,0219 | 0,0207 | -0,0500 | 0,2616 | -0,0402 | 0,0406 | 0,0831 | -0,1108 | 0,1025 | -1,5047 | 0,1256 | 0,0908 | -0,1211 | 0,1169 | 0,9120 | 0,8529 | -0,1982 | -0,0323 |
| vagina | 0,0420 | -0,1827 | 0,0992 | -0,0308 | -0,2781 | 0,0684 | -0,1793 | -0,1533 | -0,1264 | 1,0160 | -0,1949 | -0,6437 | 0,5780 | -0,0472 | 1,2325 | 1,2357 | 0,7522 | 0,0127 |
